# Supplementary material for: Potential impact of intervention strategies on COVID-19 transmission in Malawi: a mathematical modelling study
Source: BMJ Open. 2021 Jul 22;11(7):e045196. doi: 10.1136/bmjopen-2020-045196 (PMC8300555; doi:10.1136/bmjopen-2020-045196)
Supplement: Supplementary data [file bmjopen-2020-045196supp001.pdf]

Supplementary Information

<u>Prevalence of comorbidities in Malawi and China</u>

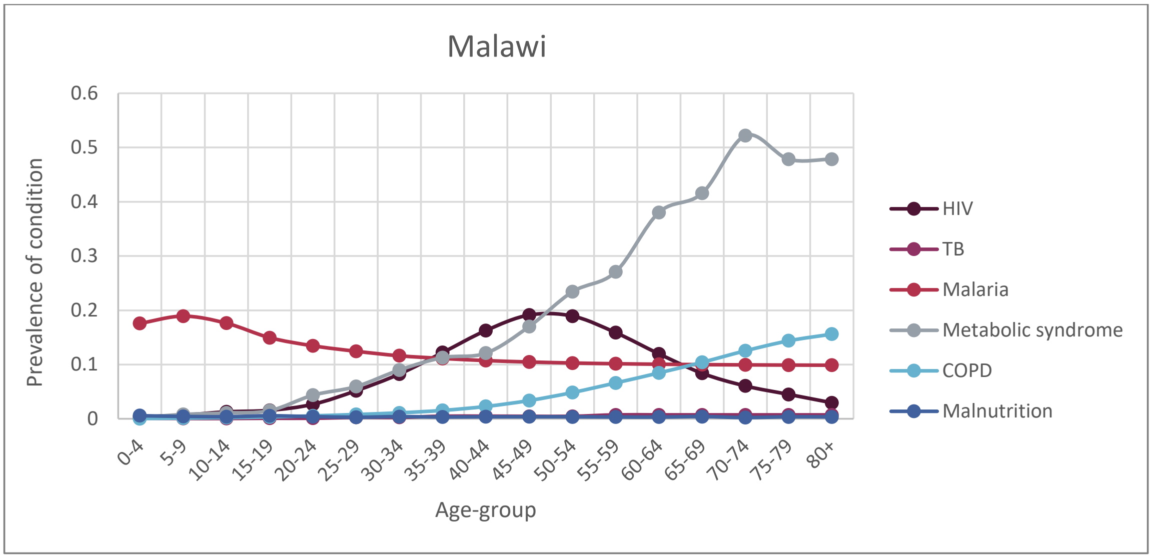

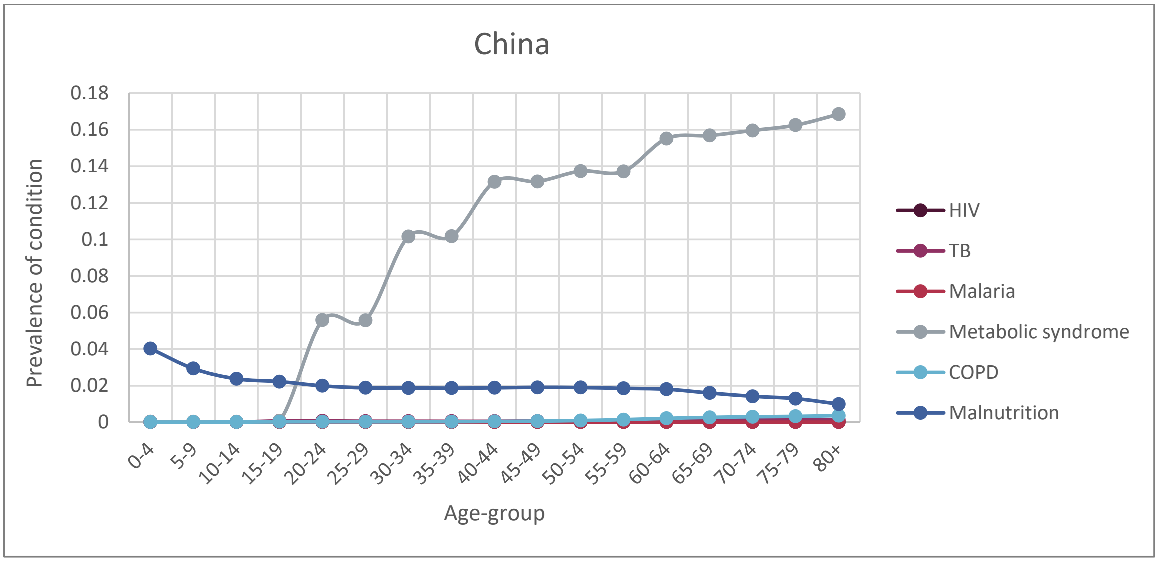

Supplementary Figure 1. Prevalence of key diseases in Malawi (top figure) and China (lower figure) used for the estimation of country-specific infection fatality ratios. Metabolic syndrome, defined in the main text and Supplementary Table 2, is a composite measure of four chronic diseases which tend to cluster in individuals.

Supplementary Table 1. Prevalence data used for the calculations of disease severity and infection fatality ratios.

**Data**	**Source**	**Notes**
TB	WHO TB Report (1)	The estimated incidence of active TB in 2019: number of active cases divided by population size for every age-group and by sex
HIV	UNAIDS Country Projections 2019 (2)<br><br>IHME (3)	For Malawi: includes all PLHIV, irrespective of treatment status. Projections are from 2019<br><br>For China and Brazil: includes all PLHIV, irrespective of treatment status, data are from 2019
Malaria	IHME (3)	For Malawi and Brazil only. Prevalence of clinical malaria episodes in 2019.
COPD	IHME (3)	For all countries, data from 2019
CVD	IHME (3)	For all countries, data from 2019<br>Included as part of "metabolic syndrome"
Diabetes	Price *et al* (4)<br><br>IHME (3)	Included as part of "metabolic syndrome"<br>Price et al 2018 for Malawi estimates and IHME (2019) for China / Brazil estimates
Hypertension	Price *et al* (4)	Included as part of "metabolic syndrome"<br>Price et al 2018 for Malawi estimates. Hypertension is included in the CVD prevalence estimates for China and Brazil.
Obesity	Global Obesity Observatory (5)	Included as part of "metabolic syndrome"<br>Malawi data are from 2016, China data are from 2015-2019, Brazil data are from 2013-2014
Malnutrition	IHME (3)	For all countries, data from 2019
Population size	UNdata (6)	For all countries, data from 2019

All disease prevalence estimates are age- and sex-stratified.

Supplementary Table 2. Parameters used in the simulation model

Parameter	Value	Notes / source
R_t	2	range 1.5-2.5
Hospital bed capacity	1.3 beds per 1,000 population	Equates to 24,869 hospital beds. Source: World Bank (7)
ICU bed capacity	25	
Mean latent period	4.6 days	Estimated at 5.1 days. The last 0.5 days are incorporated in the infectious periods to capture pre-symptomatic infectivity
Mean duration of mild infection	2.1 days	Incorporates 0.5 days of infectiousness prior to symptoms. In combination with mean duration of severe illness this gives a mean serial interval of 6.75 days
Mean duration of severe infection prior to hospitalisation	4.5 days	Mean onset-to-admission of 4 days based on unpublished analysis of data from the ICNARC study. Includes 0.5 days of infectiousness prior to symptom onset
Mean duration of hospitalisation for non-critical cases if survive	9.5 days	Based on unpublished analysis of data from the ICNARC study
Mean duration of hospitalisation for non-critical cases if die	7.6 days	Based on unpublished analysis of data from the ICNARC study
Mean duration in ICU if survive	11.3 days	Based on data from the ICNARC study adjusted for censoring
Mean duration in ICU if die	10.1 days	Based on data from the ICNARC study adjusted for censoring.
Mean duration in recovery after ICU	3.4 days	Based on unpublished analysis of data from the ICNARC study
Relative risk of mortality for patients with HIV*	1.70 – 2.70	Samples are drawn from the uniform distribution using the range defined as lower and upper bounds (8)
Relative risk of mortality for patients with TB	1.81 – 4.04	Samples are drawn from the uniform distribution using the range defined as lower and upper bounds (8)
Relative risk of mortality for patients with malaria	1.0 – 3.0	Assumed values. Samples are drawn from the uniform distribution using the range defined as lower and upper bounds
Relative risk of mortality for patients with COPD	0.77 – 4.24	Samples are drawn from the uniform distribution using the range defined as lower and upper bounds (9, 10)
Relative risk of mortality for patients with malnutrition	1.0 – 3.0	Assumed values. Samples are drawn from the uniform distribution using the range defined as lower and upper bounds
Relative risk of mortality for patients with “metabolic syndrome”	1.02 – 5.26	Samples are drawn from the uniform distribution using the range defined as lower and upper bounds. The range is defined using the lower and upper limits from the estimated values below.
Relative risk of mortality for patients with cardiovascular disease	1.20 – 5.26	Used to inform the combined “metabolic syndrome” risk (9, 10)
Relative risk of mortality for patients with hypertension	1.09 – 1.57	Used to inform the combined “metabolic syndrome” risk (8)
Relative risk of mortality for patients with obesity	1.10 – 4.34	Used to inform the combined “metabolic syndrome” risk (9)
Relative risk of mortality for patients with diabetes	1.02 – 2.84	Used to inform the combined “metabolic syndrome” risk (9, 10)

*Relative risks for HIV refer to unsuppressed and suppressed HIV infections. Where references are not cited, we use the default parameters from the COVID-19 Model of Walker *et al*.(11)

Estimates of disease severity

Following Walker *et al*., we distinguish three levels of disease severity for COVID-19: (i) those that do not require hospitalisation; (ii) those that do require hospitalisation but not intensive care; (iii) those that require intensive care (ICU).(11) Two parameters govern the proportions in each category: the proportion of infected cases requiring hospitalisation (*p_severe*) and the proportion of hospitalised cases requiring ICU (*p_critical*). We use the distributions of sampled adjusted IFR for Malawi, detailed in Methods Section 1 (i) which assume no health system constraints, as bounds for rejection sampling to determine reasonable values for these two parameters.

We sampled from a credible range for each parameter (detailed in Supplementary Table 3) based on values observed in high-income settings, keeping the age-distribution consistent with that reported in Verity *et al* and Walker *et al*.(11, 12) As we have based the calculation of IFR so far on data from China, we assumed that all persons that needed hospitalisation or intensive care received it, and that the proportion of those requiring intensive care that die, *m*, is 50%. This is a generalised assumption which incorporates both the availability of treatment and the mortality rates across both the untreated and treated critical cases. We assume no mortality in severe cases in this instance, although this assumption is changed in the simulation model. Combining the assumptions together, gives an overall IFR across the 17 age-groups as follows, where $prop_a$ is the proportion of the population in age-group *a*:

$$IFR_h = \sum_{a=1}^{17} \left(p\_severe_{h,a} * p\_critical_{h,a} * m\right) * prop_a$$

Parameter sets for *p_severe* and *p_critical* were accepted if the resulting overall IFR fell within the 95% uncertainty interval of the adjusted IFR for Malawi and the process was repeated until 1000 accepted parameter sets were generated. The resulting parameter sets were used as inputs to the epidemic simulation model.

Supplementary Table 3. Estimates of disease severity for the Malawian population

**Age-group (years)**	**Proportion cases requiring hospitalisation**		**Proportion hospitalised cases requiring ICU**	
	Credible range	Median accepted values (2.5th and 97.5th quantiles) *	Credible range	Median accepted values (2.5th and 97.5th quantiles) *
0-4	0.0002-0.0050	0.0013 (0.0005 – 0.0029)	0.0100-0.2500	0.0636 (0.0222 – 0.1456)
5-9	0.0002-0.0050	0.0013 (0.0005 – 0.0029)	0.0100-0.2500	0.0636 (0.0222 – 0.1456)
10-14	0.0002-0.0050	0.0013 (0.0005 – 0.0029)	0.0100-0.2500	0.0636 (0.0222 – 0.1456)
15-19	0.0004-0.0100	0.0027 (0.0010 – 0.0058)	0.0100-0.2500	0.0636 (0.0222 – 0.1456)
20-24	0.0010-0.0250	0.0067 (0.0026 – 0.0145)	0.0100-0.2500	0.0636 (0.0222 – 0.1456)
25-29	0.0020-0.0500	0.0134 (0.0051 – 0.0290)	0.0100-0.2500	0.0636 (0.0222 – 0.1456)
30-34	0.0032-0.0800	0.0215 (0.0082 – 0.0464)	0.0100-0.2500	0.0636 (0.0222 – 0.1456)
35-39	0.0046-0.1150	0.0309 (0.0118 – 0.0668)	0.0106-0.2650	0.0674 (0.0236 – 0.1543)
40-44	0.0058-0.1450	0.0389 (0.0148 – 0.0842)	0.0120-0.3000	0.0763 (0.0267 – 0.1747)
45-49	0.0078-0.1950	0.0523 (0.0200 – 0.1132)	0.0150-0.3750	0.0954 (0.0334 – 0.2183)
50-54	0.0116-0.2900	0.0778 (0.0297 – 0.1684)	0.0208-0.5200	0.1323 (0.0463 – 0.3028)
55-59	0.0144-0.3600	0.0966 (0.0369 – 0.2090)	0.0298-0.7450	0.1895 (0.0663 – 0.4338)
60-64	0.0204-0.5100	0.1369 (0.0522 – 0.2961)	0.0448-1.1200	0.2849 (0.0996 – 0.6521)
65-69	0.0234-0.5850	0.1570 (0.0599 – 0.3396)	0.0614-1.5350	0.3905 (0.1366 – 0.8938)
70-74	0.0292-0.7300	0.1960 (0.0747 – 0.4238)	0.0772-1.9300	0.4909 (0.1717 – 1.0000)
75-79	0.0354-0.8850	0.2376 (0.0906 – 0.5138)	0.0922-2.3050	0.5863 (0.2051 – 1.0000)
80+	0.0360-0.9000	0.2416 (0.0921 – 0.5225)	0.1418-3.5450	0.9018 (0.3154 – 1.000)

* distributions obtained through rejection sampling.

Supplementary Table 4. Social contact matrix between age-groups in Zimbabwe

Age-group	**0-4**	**5-9**	**10-14**	**15-19**	**20-24**	**25-29**	**30-34**	**35-39**	**40-44**	**45-49**	**50-54**	**55-59**	**60-64**	**65-69**	**70-74**	**75+**
**0-4**	1.006	1.030	0.883	0.732	0.647	0.712	0.645	0.463	0.289	0.214	0.211	0.215	0.179	0.121	0.075	0.048
**5-9**	0.807	2.863	2.668	0.968	0.423	0.449	0.468	0.416	0.289	0.212	0.182	0.186	0.177	0.140	0.093	0.066
**10-14**	0.659	2.544	3.766	1.865	0.580	0.413	0.452	0.536	0.375	0.251	0.207	0.208	0.193	0.177	0.136	0.096
**15-19**	0.617	1.041	2.104	2.548	1.070	0.575	0.536	0.645	0.441	0.297	0.267	0.264	0.229	0.225	0.182	0.112
**20-24**	0.947	0.790	1.135	1.858	1.363	0.969	0.752	0.723	0.474	0.363	0.333	0.310	0.281	0.254	0.199	0.123
**25-29**	1.091	0.878	0.848	1.046	1.014	1.131	0.885	0.707	0.425	0.330	0.297	0.299	0.271	0.225	0.153	0.101
**30-34**	1.268	1.175	1.191	1.252	1.010	1.136	1.119	1.032	0.593	0.405	0.356	0.364	0.341	0.249	0.164	0.102
**35-39**	0.976	1.119	1.512	1.612	1.041	0.972	1.105	1.221	0.756	0.485	0.420	0.409	0.358	0.276	0.177	0.105
**40-44**	0.960	1.225	1.668	1.738	1.076	0.923	1.001	1.192	0.863	0.629	0.488	0.434	0.378	0.311	0.207	0.117
**45-49**	0.796	1.007	1.248	1.312	0.922	0.801	0.766	0.856	0.704	0.549	0.446	0.383	0.352	0.298	0.215	0.120
**50-54**	0.844	0.928	1.106	1.266	0.909	0.774	0.722	0.796	0.587	0.479	0.417	0.407	0.397	0.331	0.242	0.159
**55-59**	0.902	0.993	1.163	1.312	0.888	0.818	0.776	0.812	0.547	0.431	0.426	0.483	0.484	0.382	0.281	0.210
**60-64**	0.823	1.041	1.191	1.250	0.882	0.815	0.797	0.781	0.524	0.435	0.457	0.532	0.567	0.449	0.347	0.259
**65-69**	0.695	1.026	1.357	1.534	0.995	0.841	0.725	0.751	0.537	0.459	0.475	0.524	0.560	0.471	0.372	0.333
**70-74**	0.592	0.943	1.442	1.712	1.080	0.791	0.664	0.668	0.495	0.458	0.481	0.532	0.599	0.515	0.455	0.383
**75+**	0.557	0.974	1.485	1.530	0.967	0.763	0.597	0.577	0.406	0.373	0.458	0.579	0.648	0.669	0.556	0.400

Values are estimated number of contacts between persons in each age-group per day. The contact matrix is adjusted to give age-specific contact rates.(13)

Supplementary Table 5. Age-dependent mortality rates in severe and critical cases.

	**Severe cases**		**Critical cases**	
Age-group	Probability death with treatment	Probability death no treatment	Probability death with treatment	Probability death no treatment
0-4	0.013	0.6	0.5	0.95
5-9	0.013	0.6	0.5	0.95
10-14	0.013	0.6	0.5	0.95
15-19	0.013	0.6	0.5	0.95
20-24	0.013	0.6	0.5	0.95
25-29	0.013	0.6	0.5	0.95
30-34	0.013	0.6	0.5	0.95
35-39	0.013	0.6	0.5	0.95
40-44	0.015	0.6	0.5	0.95
45-49	0.019	0.6	0.5	0.95
50-54	0.027	0.6	0.5	0.95
55-59	0.042	0.6	0.5	0.95
60-64	0.069	0.6	0.5	0.95
65-69	0.105	0.6	0.5	0.95
70-74	0.149	0.6	0.5	0.95
75-79	0.203	0.6	0.5	0.95
80+	0.580	0.6	0.5	0.95

The probabilities of death are the default values used in the COVID-19 Global Model of Walker *et al*.(11)

Values were derived using data from the ICNARC study in the UK.(14)

Estimates of infection fatality ratios in Malawi

The approach detailed in the main manuscript, using age-specific infection fatality ratios (IFR) from China and adjusting for the demography and comorbidity prevalence in Malawi, was repeated using estimates of IFR from Brazil to determine whether the choice of primary data on which to base this analysis significantly impacted the predicted values for Malawi.(15) The figure below (Supplementary Figure 1) shows the adjusted estimates of IFR by age in a theoretical population with no comorbidities using the data reported from China early in the pandemic and the more recent model estimated values from Brazil which incorporate seroreversion.(12) The median adjusted IFR for a population with no comorbidities is slightly higher when using the Brazil data, although the uncertainty intervals overlap for every age-group. When using the Brazil data, the adjusted population-weighted IFR for Malawi assuming no health system constraints is 0.48% (95% uncertainty interval [UI] 0.33 – 0.64%), compared with 0.26% (95% UI 0.12 – 0.69%) when using the Chinese data.

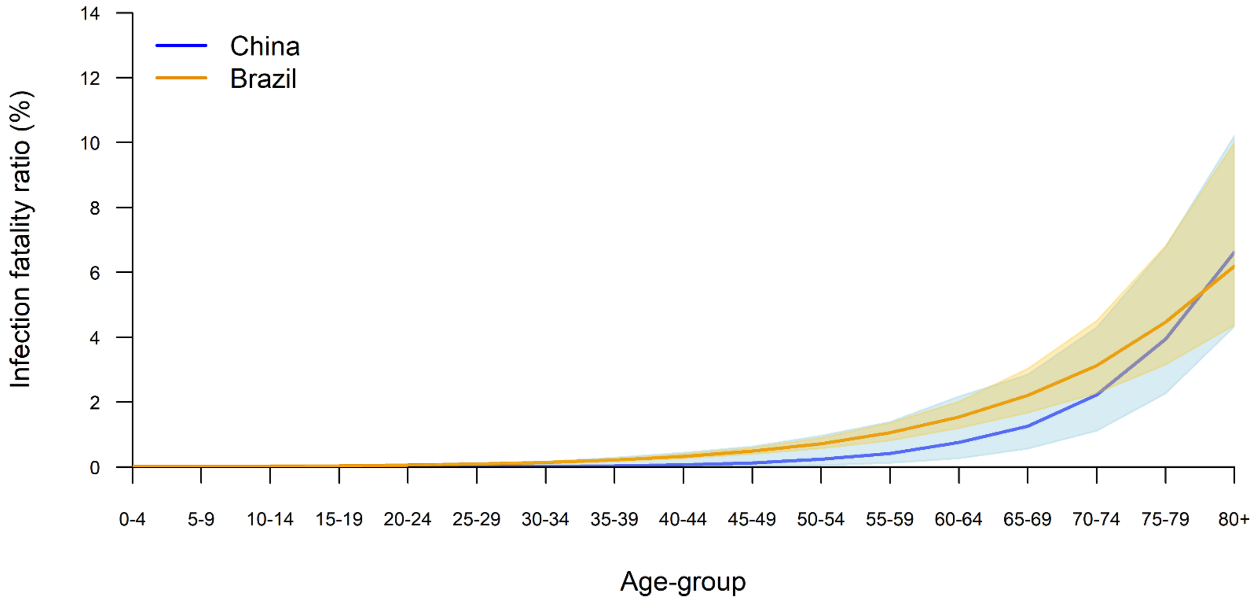

Supplementary Figure 2. Infection fatality ratios in a theoretical population assuming no comorbidities. Estimates are derived from data reported for China (blue) and Brazil (orange).

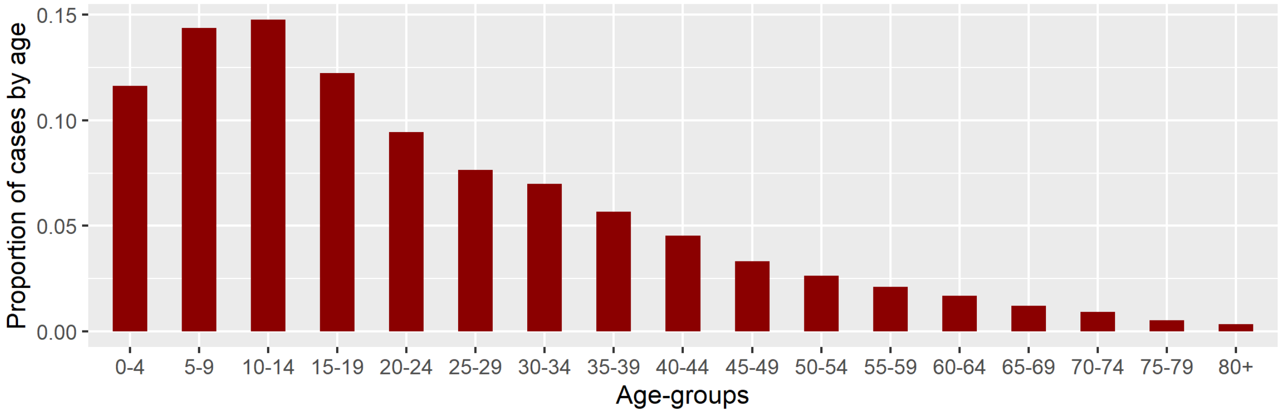

Supplementary Figure 3. Age distribution of infected cases (asymptomatic and symptomatic) at the peak of the projected unmitigated epidemic.

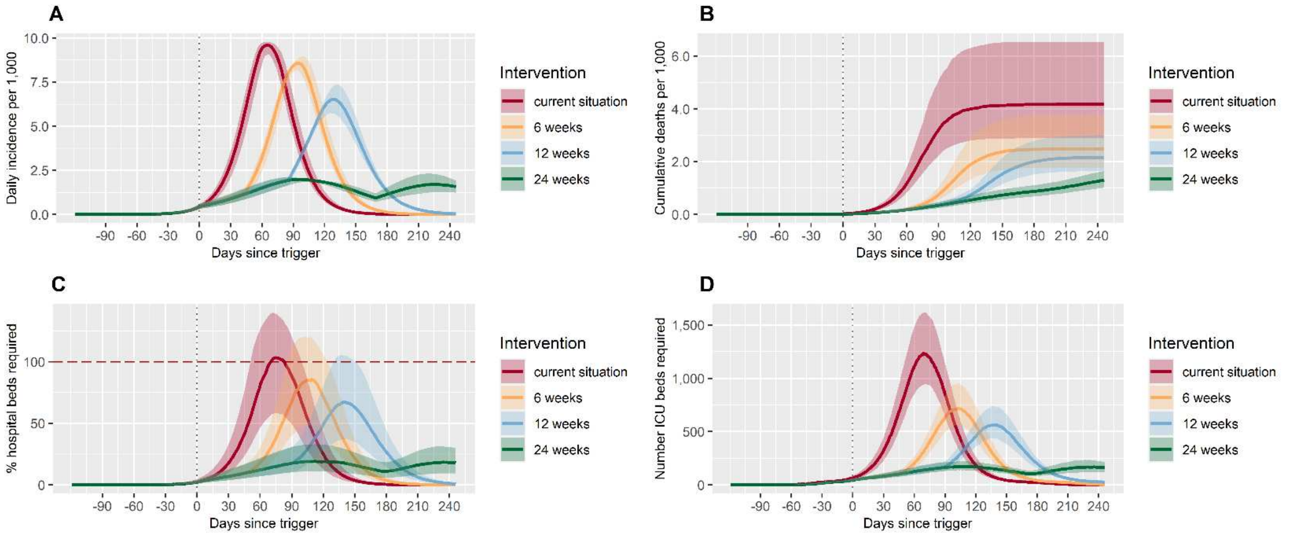

Supplementary Figure 4. Impact of changing the duration of lockdown compared with the current situation on the daily incidence per 1,000 population (A), the cumulative deaths per 1,000 population (B), the percentage of hospital beds that are required (C) and the number of ICU beds that are required (D). The current situation reflects the non-pharmaceutical interventions adopted by Malawi at the start of the second wave. Each intervention represents the implementation of lockdown over 6, 12 or 24 weeks. The trigger date (1 death per 100,000 population per week) is shown with a vertical grey dashed line. The red horizontal dashed line shows the capacity of the health system for non-intensive care (C).

Supplementary Table 6. Simulation outputs under different assumptions of R_0.

		**Baseline**	**Current situation**	**Enhanced shielding**	**Lockdown**
R_t **= 1.5**	Total cases / 1,000	563.1<br>(469.8 - 657.9)	21.7<br>(11 - 33.9)	19.6<br>(9.6 - 31.7)	18<br>(9.3 - 28.9)
	Number hospital beds required at peak	25,100<br>(13,700 – 33,800)	1,000<br>(500 – 2,000)	700<br>(300 – 1,400)	700<br>(300 – 1,400)
	Number ICU beds required at peak	1,200<br>(900 – 1,500)	100<br>(100 - 100)	100<br>(0 - 100)	100<br>(0 - 100)
	Total number of deaths / 1000	4<br>(2.8 - 6.3)	0.1<br>(0.1 - 0.2)	0.1<br>(0 - 0.2)	0.1<br>(0 - 0.2)
R_t **= 2.0**	Total cases / 1,000	769.7<br>(668.4 – 872.6)	575.3<br>(483.1 – 667.4)	499.8<br>(412.4 – 591.4)	275.0<br>(223.6 – 326.9)
	Number hospital beds required at peak	40,700<br>(28,000 – 59,700)	25,700<br>(14,500 – 34,800)	17,300<br>(9,800 – 26,400)	5,700<br>(3,100 – 9,900)
	Number ICU beds required at peak	2,600<br>(2,000 – 3,400)	1,200<br>(900 – 1,600)	600<br>(400 – 700)	300<br>(200 – 400)
	Total number of deaths / 1000	7.0<br>(4.3 – 11.6)	4.2<br>(2.9 – 6.5)	2.1<br>(1.6 – 2.9)	1.8<br>(1.4 – 2.2)
R_t **= 2.5**	Total cases / 1,000	867.8<br>(764.3 - 972.6)	740.8<br>(638.8 - 843.1)	689.4<br>(593.5 - 788.6)	535.5<br>(458.5 - 613.6)
	Number hospital beds required at peak	53,300<br>(35,000 – 80,200)	37,900<br>(26,500 – 54,800)	31,700<br>(21,600 – 43,000)	23,100<br>(12,500 – 31,800)
	Number ICU beds required at peak	3,700<br>(2,900 – 5,000)	2,300<br>(1,800 – 3,100)	1,300<br>(1,000 – 1,700)	1,100<br>(800 – 1,400)
	Total number of deaths / 1000	8.7<br>(5.4 - 14)	6.5<br>(4.0 - 10.9)	4.0<br>(2.6 – 7.0)	3.7<br>(2.7 - 5.6)

All values are medians of 1000 simulations using the sampled parameter sets. Numbers of hospital beds, ICU beds and deaths are rounded to the nearest 100. The trigger day for interventions (1.0 death per 100,000 population per week) is day 341, 120, and 81 for R_t=1.5, 2.0 and 2.5 respectively.

Supplementary Table 7. Projected cumulative number of deaths with increases in hospital capacity.

**Intervention**	**Total deaths per 1,000 population**
**Current capacity**	4.2<br>(2.9-6.5)
**Increase capacity by 25%**	3.9<br>(2.8-5.7)
**Increase capacity by 50%**	3.7<br>(2.8-5.3)
**Increase capacity by 75%**	3.6<br>(2.8-5.0)
**Increase capacity by 100%**	3.5<br>(2.8-4.7)

References

1. WHO TB burden estimates [Internet]. 2020 [cited 2nd March 2021]. Available from: https://www.who.int/tb/country/data/download/en/.

2. AIDSInfo [Internet]. 2020 [cited 22nd May 2020]. Available from: https://aidsinfo.unaids.org/.

3. Global Burden of Disease Study 2019 (GBD 2019) Results [Internet]. Institute for Health Metrics and Evaluation (IHME). 2018 [cited 2nd March 2021]. Available from: http://ghdx.healthdata.org/gbd-results-tool.

4. Price AJ, Crampin AC, Amberbir A, Kayuni-Chihana N, Musicha C, Tafatatha T, et al. Prevalence of obesity, hypertension, and diabetes, and cascade of care in sub-Saharan Africa: a cross-sectional, population-based study in rural and urban Malawi. The Lancet Diabetes & endocrinology. 2018;6(3):208-22.

5. Global Obesity Observatory [Internet]. 2020 [cited 10th June 2020]. Available from: https://www.worldobesitydata.org/.

6. Population by age, sex and urban/rural residence [Internet]. 2020 [cited 22nd May 2020]. Available from: http://data.un.org/Data.aspx?d=POP&f=tableCode%3a22.

7. World Bank Open Data [Hospital beds per 1,000 people] [Internet]. 2020 [cited 16th June 2020]. Available from: https://databank.worldbank.org/home.aspx.

8. Boulle A, Davies M-A, Hussey H, Ismail M, Morden E, Vundle Z, et al. Risk factors for COVID-19 death in a population cohort study from the Western Cape Province, South Africa. Clinical infectious diseases. 2020;Online ahead of print.

9. Noor FM, Islam MM. Prevalence and Associated Risk Factors of Mortality Among COVID-19 Patients: A Meta-Analysis. Journal of Community Health. 2020;45(6):1270-82.

10. Ssentongo P, Ssentongo AE, Heilbrunn ES, Ba DM, Chinchilli VM. Association of cardiovascular disease and 10 other pre-existing comorbidities with COVID-19 mortality: A systematic review and meta-analysis. PloS one. 2020;15(8):e0238215.

11. Walker PGT, Whittaker C, Watson OJ, Baguelin M, Winskill P, Hamlet A, et al. The impact of COVID-19 and strategies for mitigation and suppression in low- and middle-income countries. Science. 2020;369(6502):413-22.

12. Verity R, Okell LC, Dorigatti I, Winskill P, Whittaker C, Imai N, et al. Estimates of the severity of coronavirus disease 2019: a model-based analysis. The Lancet Infectious Diseases. 2020;20(6):669-77.

13. Melegaro A, Del Fava E, Poletti P, Merler S, Nyamukapa C, Williams J, et al. Social contact structures and time use patterns in the Manicaland Province of Zimbabwe. PloS one. 2017;12(1).

14. Intensive Care National Audit & Research Centre. ICNARC report of COVID-19 in critical care2020 1st July 2020. Available from: https://www.icnarc.org/.

15. Brazeau N, Verity R, Jenks S, Fu H, Whittaker C, Winskill P, et al. Report 34: COVID-19 infection fatality ratio: estimates from seroprevalence. 2020.
