# Supplementary material for: Potential impact of intervention strategies on COVID-19 transmission in Malawi: a mathematical modelling study
Source: BMJ Open. 2021 Jul 22;11(7):e045196. doi: 10.1136/bmjopen-2020-045196 (PMC8300555; doi:10.1136/bmjopen-2020-045196)
Supplement: Author's manuscript [file bmjopen-2020-045196.draft_revisions.pdf]

BMJ Open is committed to open peer review. As part of this commitment we make the peer review history of every article we publish publicly available.

When an article is published we post the peer reviewers' comments and the authors' responses online. We also post the versions of the paper that were used during peer review. These are the versions that the peer review comments apply to.

The versions of the paper that follow are the versions that were submitted during the peer review process. They are not the versions of record or the final published versions. They should not be cited or distributed as the published version of this manuscript.

BMJ Open is an open access journal and the full, final, typeset and author-corrected version of record of the manuscript is available on our site with no access controls, subscription charges or pay-per-view fees (http://bmjopen.bmj.com).

If you have any questions on BMJ Open's open peer review process please email info.bmjopen@bmj.com

BMJ Open

The potential impact of intervention strategies on COVID-19 transmission in Malawi: A mathematical modelling study

Journal:	*BMJ Open*
Manuscript ID	bmjopen-2020-045196
Article Type:	Original research
Date Submitted by the Author:	24-Sep-2020

Complete List of Authors:	Mangal, Tara; Imperial College London, Infectious Disease Epidemiology<br>Whittaker, Charlie; Imperial College London, Infectious Disease Epidemiology<br>Nkhoma, Dominic; University of Malawi College of Medicine<br>Ng'ambi, Wingston; University of Malawi College of Medicine<br>Watson, Oliver; Imperial College London School of Public Health, Infectious Disease Epidemiology<br>Walker, Patrick; Imperial College London, Infectious Disease Epidemiology<br>Ghani, AC; Imperial College London, Infectious Disease Epidemiology<br>Revill, Paul; University of York, Centre for Health Economics<br>Colbourn, Timothy; UCL Institute for Global Health,<br>Phillips, Andrew; UCL, HIV Epidemiology and Biostatistics Group<br>Hallett, Timothy; Imperial College London, Infectious Disease Epidemiology<br>Mfutso-Bengo, Joseph; University of Malawi College of Medicine

Keywords:	Public health < INFECTIOUS DISEASES, COVID-19, EPIDEMIOLOGY, Infection control < INFECTIOUS DISEASES

SCHOLARONE™
Manuscripts

BMJ

I, the Submitting Author has the right to grant and does grant on behalf of all authors of the Work (as defined in the below author licence), an exclusive licence and/or a non-exclusive licence for contributions from authors who are: i) UK Crown employees; ii) where BMJ has agreed a CC-BY licence shall apply, and/or iii) in accordance with the terms applicable for US Federal Government officers or employees acting as part of their official duties; on a worldwide, perpetual, irrevocable, royalty-free basis to BMJ Publishing Group Ltd ("BMJ") its licensees and where the relevant Journal is co-owned by BMJ to the co-owners of the Journal, to publish the Work in this journal and any other BMJ products and to exploit all rights, as set out in our licence.

The Submitting Author accepts and understands that any supply made under these terms is made by BMJ to the Submitting Author unless you are acting as an employee on behalf of your employer or a postgraduate student of an affiliated institution which is paying any applicable article publishing charge ("APC") for Open Access articles. Where the Submitting Author wishes to make the Work available on an Open Access basis (and intends to pay the relevant APC), the terms of reuse of such Open Access shall be governed by a Creative Commons licence – details of these licences and which Creative Commons licence will apply to this Work are set out in our licence referred to above.

Other than as permitted in any relevant BMJ Author's Self Archiving Policies, I confirm this Work has not been accepted for publication elsewhere, is not being considered for publication elsewhere and does not duplicate material already published. I confirm all authors consent to publication of this Work and authorise the granting of this licence.

For peer review only

Title: The potential impact of intervention strategies on COVID-19 transmission in Malawi: A mathematical modelling study

Authors: Tara D. Mangal[1] PhD, Charlie Whittaker[1], Dominic Nkhoma[2] PhD, Wingston Ng'ambi[2], Oliver J Watson[1], Patrick Walker[1] PhD, Azra Ghani[1] PhD, Paul Revill[3] MSc, Tim Colbourn[4] PhD, Andrew Phillips[4] PhD, Timothy B. Hallett[1] PhD, Joseph Mfutso-Bengo[2] PhD

Affiliations:

[1]Medical Research Council Centre for Global Infectious Disease Analysis & Abdul Latif Jameel Institute for Disease and Emergency Analytics, Imperial College London, London, UK

[2]Health Economics & Policy Unit, College of Medicine, University of Malawi, Malawi

[3]Centre for Health Economics, University of York, UK

[4]Institute for Global Health, University College London, UK

Address for correspondence: Tara Mangal, Department of Infectious Disease Epidemiology, School of Public Health, Faculty of Medicine, Imperial College London, Norfolk Place, London W2 1PG; email: t.mangal@imperial.ac.uk

Keywords: SARS-CoV-2, COVID-19, Malawi, non-pharmaceutical interventions, infection fatality rate, comorbidity, face covering, severity, mortality.

For peer review only

Abstract

Background

COVID-19 mitigation strategies have been challenging to implement in resource-limited settings such as Malawi due to the potential for widespread disruption to social and economic well-being. Here we estimate the clinical severity of COVID-19 in Malawi, quantifying the potential impact of intervention strategies and increases in health system capacity.

Methods

The infection fatality ratios (IFR) in Malawi were estimated by adjusting reported IFR for China accounting for demography, the current prevalence of comorbidities and health system capacity. These estimates were input into an age-structured deterministic model, which simulated the epidemic trajectory with non-pharmaceutical interventions. The impact of a novel therapeutic agent and increases in hospital capacity and oxygen availability were explored, given different assumptions on mortality rates.

Findings

The estimated age-specific IFR in Malawi are higher than those reported for China, however the younger average age of the population results in a slightly lower population-weighted IFR (0.48%, 95% uncertainty interval [UI] 0.30% – 0.72% compared with 0.60%, 95% CI 0.4% – 1.3% in China). The current interventions implemented, (i.e. social distancing, workplace closures and public transport restrictions) could potentially avert 3,100 deaths (95% UI 1,500 – 4,500) over the course of the epidemic. Enhanced shielding of people aged ≥ 60 years could avert a further 30,500 deaths (95% UI 17,500 – 45,600) and halve ICU admissions at the peak of the outbreak. Coverage of face coverings of 60% under the assumption of 50% efficacy could be sufficient to control the epidemic. A novel therapeutic agent, which reduces mortality by 0.65 and 0.8 for severe and critical cases respectively, in combination with increasing hospital capacity could reduce projected mortality to 2.55 deaths per 1,000 population (95% UI 1.58 – 3.84).

Conclusion

For peer review only

The risks due to COVID-19 vary across settings and are influenced by age, underlying health and health system capacity.

For peer review only

Strengths and limitations of this study

- There is an urgent need to characterise how the SARS-CoV-2 outbreak could spread in Malawi and how different interventions could impact mortality and health system demands.
- This is one of the first studies to date which combines country-specific estimates of the infection fatality ratio (IFR) of COVID-19 for Malawi adjusted for comorbidity prevalence and with consideration to the prevailing health system constraints and the impact of these constraints on mortality rates.
- An age-structured deterministic model was used to characterise the spread of SARS-CoV2 throughout Malawi using the Malawi-specific adjusted IFR.
- The impacts of non-pharmaceutical interventions, novel therapeutics and hospital capacity were analysed and the effects on incidence, mortality and hospital demand are presented.
- The IFR used as the baseline, from which we infer the adjusted estimates for Malawi, and some key parameters rely on data from outside sub-Saharan Africa due to the limited numbers of cases there and may therefore not be directly transferable to Malawi.
- As case numbers rise in Malawi and further data are collected on the impacts of each intervention strategy, the model can be calibrated and tested to further refine the model predictions.

For peer review only

Author's contributions

Literature search: TDM, TC, AP, TBH

Figures: TDM

Study design: All authors

Data collection: TDM, CW, DN, WN, OJW, PW, AG, TBH, JM-B

Data analysis: TDM, TBH, AP, TC

Data interpretation: All authors

Writing: All authors

For peer review only

INTRODUCTION

As of 22nd August 2020, the novel coronavirus SARS-CoV-2 had spread throughout 216 countries, with over 21 million cases and 760,000 deaths worldwide.[1] In response to this threat, numerous African countries acted swiftly, implementing a suite of control measures aimed at slowing down the spread of disease and minimising deaths. On 20th March 2020, Malawi declared a state of national disaster, ordering all schools to close, barring large gatherings and restricting international travel. This was followed by the order of a 21-day lockdown to begin on 18th April 2020, which was later suspended by the High Court of Malawi due to concerns around the implications on vulnerable populations. Further control measures were introduced in April, including social distancing and restrictions on public transport use. The first three cases in Malawi were confirmed on 2nd April 2020 and by 8th July 2020, clusters of cases had occurred across 28 districts (1,818 confirmed cases with 19 deaths) although widespread community transmission had not been reported.[1,2]

Given the limited capacity of the health system in Malawi to respond to an emerging outbreak and the likelihood that effective pharmaceutical interventions may not be available for many months, there is a critical reliance on non-pharmaceutical interventions (NPI) to reduce transmission. These measures include isolation of suspected/confirmed cases, contact tracing, social distancing, travel restrictions, face covering, school and workplace closures and shielding of the most vulnerable.[3-6]

The impact of NPI can be summarised as a change in the effective reproduction number *Rt*, which represents the average number of secondary infections resulting from one infected case. Strict interventions such as lockdown, where population movement is limited to only essential travel and most public facilities and transport links are closed, have shown the most success in reducing transmission.[5,7] However major restrictions to working practices or public transport may have catastrophic implications in Sub-Saharan Africa, where many have limited financial capacity to withstand income shocks and no access to social protection programmes.[8] Face coverings have been recommended by WHO as one possible intervention which could reduce transmission of SARS-CoV-2 with minimal socioeconomic implications despite a lack of high-quality evidence.[9,10] Nevertheless, several countries, (including, Canada, South Korea and Czech Republic), have made face

For peer review only

coverings mandatory in public spaces and the UK has made the use of face masks mandatory on public transport and in shops.

The impact of NPI on SARS-CoV-2 transmission in Malawi depends critically on the local context such as population behaviour (including uptake of and compliance with such measures), population movement and contact patterns (Malawi is over 80% rural and many rely on subsistence farming) and health system capacity.[11]

A key priority during this emerging pandemic, is estimating clinical severity and health system requirements. Current oxygen capacity in hospitals may not be sufficient to give supportive care to large numbers of severe COVID-19 cases; 65% of 34 hospital wards in Malawian hospitals recently assessed had (what was considered pre-COVID-19) adequate access to oxygen and priority is now being given to scaling up access and supplies urgently.[12,13] The impact of oxygen supplementation on COVID-19 mortality is not well established however early studies suggest oxygen could reduce the need for mechanical ventilation and lower the risk of death.[14] Several therapeutic agents have been considered for treatment of severe COVID-19, two of which, Dexamethasone and Remdesivir, have been effective in improving patient outcomes.[15,16]

Infection fatality ratios (IFR), defined as the number of deaths divided by the number of infections are challenging to estimate, particularly in an emerging outbreak, due to the difficulties in identifying the true number of infected people (both asymptomatic and symptomatic). IFR are strongly dependent on age and the majority of deaths reported early in the epidemic were among those aged over 60 years.[17-19] There is also now a growing body of evidence on the elevated risk of mortality with certain underlying comorbidities, such as cardiovascular disease (CVD), diabetes, chronic obstructive pulmonary disease (COPD) and infectious diseases, for example HIV, TB and malaria.[20-23] The majority of these data were reported from high-income settings, which have borne the highest burden of disease recorded so far. It is not yet clear how these risk factors will affect disease severity in countries like Malawi, which have a younger population overall, but a high prevalence of infectious diseases and untreated chronic conditions.

Objectives

For peer review only

Three objectives form the focus of this paper: 1) estimate disease severity caused by SARS-CoV-2 in the Malawian population given its demographic structure, the prevalence of key comorbidities and health system capacity; 2) examine the potential impact of a range of NPI that have been or could be used in Malawi; 3) on that basis, investigate the potential extent to which increasing health system capacity and/or providing therapeutics could contribute to reducing deaths due to SARS-CoV-2 infection.

For peer review only

METHODS

We present the methods in three sections that relate to each of our aims.

1. Estimates of Infection Fatality Ratios in Malawi

Our approach utilises data on age-specific IFR from China (one of the few studies which applies demography-adjusted under-ascertainment corrections) and then makes adjustments based on the relative burdens of diseases relevant to COVID-19 risk between China and Malawi, making assumptions about the extent to which each disease affects IFR.[19]

The prevalence of HIV (virally suppressed and unsuppressed), active TB, clinical malaria, CVD, COPD, hypertension, diabetes (type I and II), obesity (defined as BMI ≥28 kg/m^2 according to Chinese criteria and ≥30 kg/m^2 using Malawian criteria) and malnutrition were extracted for Chinese and Malawian populations (see Supplementary Table 1 for data sources). We created a unified risk factor for "metabolic syndrome", the prevalence for which was the greatest of the prevalences of CVD, hypertension, obesity and diabetes due to clustering of these conditions. The risk for metabolic syndrome was taken as the highest risk reported for any of the pooled conditions. Given the considerable uncertainty in these estimates along with likely differences across settings, we considered a wide range of relative risk values for each comorbidity (Supplementary Table 2).

The age-distributed estimates for IFR in Malawi were derived from those published by Verity *et al* for cases reported in mainland China, using linear interpolation on the log scale to derive values in five-year age-groups from the 10-year age-bands reported.[19] Adjusted IFR estimates by age for Malawi were computed as follows:

1) Lognormal distributions were derived for each of the age-distributed IFR from China such that the mean matched the mean IFR and 95% of the probability mass fell inside the reported 95% bounds. Uniform distributions were defined for the relative risks of mortality due to COVID-19 for each comorbidity covering the range described in Supplementary Table 2
2) Age-specific IFR and relative risks were sampled from the defined distributions

For peer review only

3) Age-distributed IFR for a theoretical population with no comorbidities (IFR^*_a) were computed using Equation 1:

$$IFR^*_a = \frac{IFR_{h=China,a}}{\sum_i (r_i . c_{i,h,a})} \qquad \text{Equation 1}$$

Where $IFR_{h=China,a}$ is the sampled IFR in setting h (where h is China), i is the index for each comorbidity, r_i is the sampled relative risk of mortality for each condition and $c_{i,h,a}$ is the prevalence of each comorbidity in setting h. All terms except relative risk values are additionally indexed by age-group a.

4) The adjusted IFR for Malawi were then estimated using Equation 2:

$$IFR_{h=Malawi,a} = IFR^*_a \sum_i (r_i * c_{i,h=Malawi,a}) \qquad \text{Equation 2}$$

5) Steps 2-4 were repeated 1000 times and the median adjusted IFR and uncertainty intervals were calculated as the 50th, 2.5th and 97.5th quantiles from the sampled estimates

A summary for the average IFR for Malawi was obtained by weighting the age-specific IFR by the proportion of the population in each age-group, as follows:

$$IFR_{h=Malawi} = \frac{\sum_a (N_a . IFR_{h=Malawi,a})}{\sum_a N_a} \qquad \text{Equation 3}$$

Where N_a is the number of persons in that age-group.

This method assumed that the differences between IFR in Malawi compared with China are due to comorbidities and age-structure: the effect of access to and quality of healthcare was accounted for through the following additional steps:

1) Parameter sets defining the proportion of COVID-19 cases requiring different levels of hospital care (severe or critical) were generated using rejection sampling on the basis of (i) prior information from high-income settings; (ii) assumptions for the mortality according to disease severity with treatment;

For peer review only

and, (iii) accepting those parameter sets which resulted in an IFR which fell within the IFR computed for this setting under the assumption of similar healthcare capacity.

2) These estimated proportions of disease severity were used in the simulation model, with additional assumptions about mortality in the absence of care (severe cases experience an age-dependent mortality rate which is doubled if no treatment is received; mortality rates are 0.5 for critical cases receiving treatment and 0.95 if untreated).
3) Epidemic trajectories were simulated in which the availability of hospital care is limited to the level currently prevailing in Malawi. The induced overall IFR in Malawi given the current healthcare system constraints at the end of the epidemic is "Number died of COVID-19 / Number ever infected with SARS-CoV-2".

Further details are provided in the SI.

2. Estimates of the Potential Impact of NPI on Transmission of SARS-CoV-2

The COVID-19 Model of Walker *et al.* was used to make projections of the spread of SARS-CoV-2 in Malawi under a number of NPI scenarios.[24] Briefly, the model comprises an age-structured compartmental deterministic framework which describes the transmission of SARS-CoV-2 through an otherwise homogenous population. The rates of contact between age-groups are derived from the Manicaland study in Zimbabwe (Supplementary Table 4).[25] Infected (and infectious) cases are classified as mild (not requiring care), severe (requiring hospitalisation / oxygen) and critical (requiring ICU / mechanical ventilation) with the likelihood of receiving care constrained by the prevailing health system capacity. We assume that not all critical cases will require mechanical ventilation, but for those that do, access to an ICU bed will also indicate availability of mechanical ventilation. In each infected stage, there is a probability of death, derived from multinational analyses from data in China, UK and US. It is assumed that hospitalised cases will not contribute to transmission. In the case of a person needing care but health system capacity being exhausted, the person is exposed to a risk of death consistent with no care being received (Supplementary Table 3). The probability of death in severe cases not

For peer review only

receiving treatment is lowered from the default values in the Walker *et al* model to double that of treated cases to reflect the low mortality observed in Malawi to date.[26]

We assumed R_0 = 2, which is a central value for the estimates of R_0 in Malawi before nationwide non-pharmaceutical interventions were introduced and vary this between 1.5 and 3 to reflect the uncertainty in this assumption.[27] At the start of the outbreak, 20 cases were seeded in age-groups 35-54 reflecting the ages of those who are most likely to have travelled internationally and acquired infection.

We ran each simulation 1000 times over 250 modelled days (365 days when changing the duration of NPI) using the sampled parameter sets for disease severity (see above). The outcomes of each intervention on the daily numbers infected, health system requirements (broken down by severity) and deaths are presented.

Non-pharmaceutical intervention strategies

The interventions under consideration are shown in Supplementary Table 4. The duration of lockdown was varied between 6 and 24 weeks, whereupon the previously implemented intervention strategies were resumed.

Face coverings were analysed as an incremental intervention on top of the existing measures and we explored a full range of values for efficacy and proper usage assuming that the current measures in place remain for the duration of the simulation. We don't distinguish between household and non-household transmission and assume adherence and efficacy jointly reduce the risk of transmission to the whole population.

The low numbers of deaths reported to date in Malawi coupled with the high potential for under-reporting make formal calibration to surveillance data problematic. We opt, therefore, to present a hypothetical future scenario where community transmission has started to occur.

3. Estimates of the impact of increasing health system capacity

The projected effects of increasing the number of non-intensive care hospital beds plus availability of oxygen and the introduction of a novel therapeutic agent were examined assuming that the current intervention strategies would remain in place indefinitely. We simulated an increase in hospital bed capacity (plus access to

For peer review only

oxygen) by up to 100% from the trigger day of 1.0 deaths per 100,000 population, presenting the resulting impact on the cumulative number of deaths projected to occur over the epidemic, which depends critically on the assumptions around mortality in severe cases. We conservatively estimated age-dependent mortality rates would double if severe cases were untreated but given the uncertainty in this, explored an additional scenario in which the untreated mortality rate was 0.6 for all age-groups.[24] Additionally, the impact of a novel therapeutic agent was analysed, assuming a proportional reduction in mortality for severe and critical cases (0.65 and 0.8 respectively, based on the findings from the RECOVERY trial on Dexamethasone) applicable to all age-groups. We assumed that the therapeutic agent could be administered to those in need even if hospital beds or ICU beds were not available.

All analyses were conducted in R statistical software, version 3.6.3 (https://www.r-project.org/).

Data sharing agreement

Source code and supporting documentation for the SEIR model is available at https://github.com/mrc-ide/squire. All data used in the analyses are publicly available and sources have been listed for each.

Patient and Public Involvement

Patients and/or the public were not involved in the design, or conduct, or reporting, or dissemination plans of this research.

Role of the funding source

The funders of this study had no role in study design, data analysis, data interpretation or writing of the report. All authors had access to all data in the study and accept final responsibility for the decision to submit for publication.

For peer review only

RESULTS

1. Estimates of the infection fatality rate in Malawi

The estimated age-specific IFR for Malawi are higher for every age-group than those reported in China under the assumption of similar healthcare availability (Figure 1). However, the estimated population-weighted IFR are lower in Malawi (IFR 0.24%, 95% uncertainty interval [UI] 0.12 – 0.61%, compared with 0.6%, 95% confidence interval 0.40% – 1.30% in China) due to the younger average age of the population. When incorporating the known health system capacity constraints in Malawi through the simulation model, overall population-weighted IFR increase but remain slightly lower than the estimates for China although the difference is not significant (overall IFR 0.48%, 95% UI 0.30% – 0.72%).

2. Estimates of the potential impact of NPI on transmission of SARS-CoV-2

The projected unmitigated scenario is presented as a counterfactual, showing what could occur had no interventions been introduced (Figure 2). With the current interventions in place, and assumed to be in place indefinitely, we estimate approximately 3,100 deaths (95% UI 1,500 – 4,500) could be averted over the course of the epidemic compared with an unmitigated scenario in which 80,400 deaths (95% UI 49,500 – 118,700) are projected to occur (Table 1). The projected death rate over the 250 modelled days with the current interventions in place is 4.20 deaths per 1,000 population (95% UI 2.51-6.00 deaths per 1,000 population). These measures would also delay the spread of infection, shifting the peak in infections by approximately 11 days.

The predicted age distribution of infected people at the peak of the epidemic shows that the majority of infections occur in the younger ages (<20 years) which make up >50% of the population and have high contact rates (Supplementary Figure 1), although only 1.3% of deaths occur in that group. There is, however, considerable uncertainty around the prevalence and impact of comorbidities such as HIV and malnutrition in these age-groups. The majority of projected deaths occur in those aged over 70 years.

For peer review only

Of mitigation strategies modelled, long-term lockdown has the largest impact, bringing the infection rate down to 71 infections per 1,000 population (95% UI 58– 85 per 1,000 population) and the mortality rate to 0.5 deaths per 1,000 population (95% UI 0.3-0.8 per 1,000 population, equivalent to 10,100 deaths, 95% UI 6,400 – 15,300, Table 1). Applying lockdown over 6, 12 and 24 weeks delays the peak incidence but an outbreak with a similar magnitude to the projections under the current situation would still occur after lockdown is relaxed in the absence of any further interventions (Supplementary Figure 2).

With current interventions in place, coverage of face coverings would need to exceed 60% (50% with shielding implemented simultaneously) with a minimum of 50% efficacy in order to reduce projected *Rt* to below 1 (Figure 3).

3. Estimates of the impact of increasing health system capacity

If it is assumed that mortality is low in severe cases, then increasing capacity for non-intensive care (hospital beds with oxygen availability) by 50% reduces the projected mortality rate to 3.89 deaths per 1,000 population (95% UI 2.47 – 5.79) compared with 4.04 (95% UI 2.51 – 5.98) under the current scenario (Supplementary Table 8). Doubling hospital and oxygen capacity could only marginally further reduce this to 3.79 deaths per 1,000 population (95% UI 2.45 – 5.61). If, instead, we assume that the probability of death in untreated severe cases is 0.6 across all ages (consistent with the clinical estimates used by Walker *et al* derived from high-income settings) , the proportional reduction in expected deaths would be larger, falling from 5.96 deaths per 1,000 population (95% UI 2.99 – 10.62) in the current scenario to 4.41 deaths per 1,000 population (95% UI 2.48 – 7.87) with double the hospital bed capacity.[24] Introducing a novel therapeutic agent that is capable of reducing mortality by 0.6 for severe cases by 0.85 for critical cases, in combination with a 50% scale up in hospital capacity reduces projected deaths to 2.55 deaths per 1,000 population (95% UI 1.58 – 3.84, Figure 4).

For peer review only

DISCUSSION

The results shown here give several important insights into the potential spread and severity of SARS-CoV-2 infection in Malawi and what can be done to prepare for it.

First, under the assumption of similar access to healthcare, estimated age-specific IFR in Malawi are higher compared with China although overall population-weighted IFR are lower due to the younger average age of the population.[19] As the availability of health care is much lower in Malawi, the overall expectation for IFR increases to 0.48% (95% UI 0.30% – 0.72%), slightly lower although still within the range of estimates reported across the Americas, Asia and Europe (overall IFR 0.70, 95% CI 0.57 – 0.82, range 0.28 – 0.89).[28] Estimates of mortality in Uganda follow a similar trend, with a lower predicted disease burden than the comparator regions (Europe, America and China) and the majority of risk arising indirectly due to disruptions to the health service.[29] Other studies have also reported a differential risk of severe disease in African settings compared with European countries due to underlying health conditions although there is considerable uncertainty in these analyses.[30,31]

Second, we find that the intervention strategies that have been implemented so far in Malawi would be unlikely to suppress or substantially mitigate the epidemic. Although lockdowns have been highly effective across a number of settings, it is may be impractical in Malawi for a number of reasons.[5,32,33] Approximately 50% of the Malawian population lives in poverty, meaning there is no financial buffer if people are unable to earn money.[34] The disruption to food production and delivery chains is likely to impact those who are most vulnerable, with food shortages likely to occur within days of lockdown being implemented.

In principle, shielding of the elderly (plus other vulnerable populations, such as people living with HIV or TB) should be effective in significantly reducing the death rate. The practicalities of moving elderly people into separate accommodation in the African context are uncertain at best but it is a potentially risky strategy as a single imported case could have devastating effects. In high-income countries, this strategy has proven difficult with outbreaks occurring in many nursing homes.[35] Designating shielded households, or even single rooms

For peer review only

within a household may be a viable strategy in Malawi which has an extensive network of community health workers who could facilitate and support such measures.

Given the difficult choices facing decision-makers in Malawi and elsewhere, it is not surprising that significant attention has turned to less disruptive strategies for restricting some population movement and advocating use of face coverings, a putatively low-cost and readily available means of reducing transmission risk. However, there is no clear indication as yet whether widespread proper use of face coverings would be possible, and thus it remains highly uncertain how effective face covering will be in reducing risk of SARS-CoV-2 transmission. Delaying the epidemic by even a few months could allow time for new therapeutic interventions to be implemented subject to evidence of effect.[36] Additional interventions, such as contact tracing and testing (test, trace, isolate) and local quarantining could have a significant impact on the spread of infection and have been regularly used for case-finding and containment for HIV and TB in low and middle-income countries.[37-40] However they are extremely intensive and difficult to implement at a large scale and therefore may not be a feasible option for mitigating the outbreak in Malawi.

Third, we find that increases in certain types of capacity in hospital may contribute to reducing deaths. The current priorities for COVID-19 response in Malawi are to significantly expand access to oxygen concentrators in existing hospitals through the acquisition of new concentrators or using splitters to increase capacity.[13] The expected impact of this is driven by the assumptions on the mortality of severe cases, for which very few data from low-income countries are available to inform. The potential benefit is largest when the estimated mortality in severe cases is high. Additionally, there are likely to be improvements to long-term morbidity and lung health if severe cases receive oxygen when required, combined with reduced probability of requiring mechanical ventilation, although we do not capture this here. We define individuals as mild, severe or critical from the outset and do not include a probability of progression from severe to critical, which could be reduced by hospital bed and oxygen availability. There is an urgent need for further data to analyse the longer-term impacts of COVID-19 infection to inform this. Randomised trials of treatments for COVID-19 are underway in many

For peer review only

countries and the introduction of a low-cost effective therapeutic agent, such as Dexamethasone or (generic) Remdesivir, could have the potential to significantly reduce clinical severity.[15,16,36]

The IFR that we use as our baseline along with some key parameters including treatment outcomes rely on data from mainland China, North America and Europe which may not be directly transferable to Malawi.[5,19,24] Our results are sensitive to the assumptions inherent in these analyses and cannot yet be fully parameterised using data from African settings due to the limited numbers of cases there. Age-structured contact matrices are derived from studies in Zimbabwe although we expect that there are unlikely to be significant differences between the two settings that would meaningfully affect the conclusions drawn. Household structure is not incorporated therefore we cannot adjust for increased risk of infection within households. This may be of particular importance in Malawi where households are multigenerational and there may not be space to designate separate rooms for high-risk individuals. The absence of nosocomial transmission in this model may result in an under-estimation of the incidence of SARS-CoV-2 if PPE availability within hospitals is sub-optimal. An important next step in this analysis would be the integration of the model with geographically-disaggregated surveillance data on testing, deaths, movement and other data which could capture local transmission and potentially open the way to more finely targeted interventions that may maximise epidemic control with lesser disruptions overall.

The estimates of relative risk of mortality with comorbidities are derived mainly from studies in high-income settings and only one is based on data from sub-Saharan Africa. These risks may vary by age although we do not capture this here. The management of comorbidities is likely to differ across settings and so the corresponding risk of mortality with these conditions may vary also. Lastly, we do not distinguish between oxygen requirements or mechanical ventilation needs of severe and critical cases although the mortality benefits from a novel therapeutic agent could vary between these groups.

The slow initial spread of SARS-CoV-2 up to 20th June 2020 could be consistent with a lower R_0 than is assumed here, an imperfect surveillance system with low numbers of tests being carried out, or few introductions of cases to Malawi with measures having been sufficient so far in limiting transmission.[27] This

For peer review only

study has focussed on the effects of non-pharmaceutical interventions and scaling up health system capacity in respect of one disease, COVID-19. However imposing lockdown could disrupt routine health services such as the provision of care for HIV, tuberculosis and malaria along with national immunisation programmes, compounding increases in mortality rates.[41] Balancing the competing demands on health versus economic productivity, poverty and education is an extremely difficult decision and we present these projections as a series of hypothetical scenarios which could be used to inform decision-making.

These outputs are not intended to be a forecast of what will happen in Malawi and evidence should be reviewed in the light of the continuously evolving surveillance data and combined with detailed analyses on the broader impacts of any potential interventions. Lessons could be learnt from the South African response, which implemented a rapid, phased strategy, successfully delaying the outbreak despite considerable challenges. In addition to physical distancing and restrictions on movement, South Africa capitalised on existing experienced teams of community health workers to conduct active case-finding along with redirecting contact-tracing teams, previously established for TB control, to conduct COVID-19 contact-tracing and monitor quarantine compliance.[38] Clearly lockdown would have the biggest impact on spread of SARS-CoV-2, however in settings where this is not feasible, a combination of interventions such as shielding, face covering, increasing hospital capacity and therapeutic agents could together have a significant impact on mortality.

Declaration of interests

The authors declare no conflicts of interest.

Acknowledgments

Dr Mangal, Prof Hallett, Prof Phillips, Dr Colbourn, Prof Mfutso-Bengo, Mr Revill, Dr Nkhoma and Mr Ng'ambi are supported by UK Research and Innovation as part of the Global Challenges Research Fund, grant number MR/P028004/1

For peer review only - http://bmjopen.bmj.com/site/about/guidelines.xhtml

TM, CW, PW, OJW, AG and TBH acknowledge joint Centre funding from the UK Medical Research Council and Department for International Development. Grant reference: MR/R015600/1

We would like to thank the Imperial College COVID-19 Response Team for their feedback and assistance with method development. We would also like to thank the University of Malawi Health Economics and Policy Unit Think Tank members for their support and collaboration in this project.

For peer review only

Table 1. Outputs from intervention strategies over 250 days

	Unmitigated	Current situation	Enhanced shielding	Lockdown
Total cases / 1,000	770 (666-875)	584 (507-662)	513 (436-595)	71 (58-85)
Number hospital beds required at peak	40,000 (27,800-59,900)	35,600 (25,300-53,100)	27,900 (17,800-38,000)	2,100 (1,100-3,500)
Number ICU beds required at peak	2,100 (1,400-3,400)	1,800 (1,200-2,900)	900 (600-1,400)	100 (100-200)
Total deaths / 1,000	4.2 (2.6-6.2)	4.0 (2.5-6.0)	2.4 (1.63.64)	0.5 (0.3-0.8)

All values are medians of 1000 simulations using the sampled parameter sets for disease severity. Median hospital and ICU beds are rounded to the nearest 100.

List of Figures

Figure 1. Adjusted estimates of infection fatality ratios for the Malawian population with unconstrained healthcare and constrained healthcare (according to current health system capacity) compared with estimates from China.

Figure 2. Impact of non-pharmaceutical interventions compared with a baseline (unmitigated) scenario on the daily incidence per 1,000 population (A), the cumulative deaths per 1,000 population (B), the percentage of hospital beds that are required (C) and the number of ICU beds that are required (D). The unmitigated scenario presents the counterfactual situation had no interventions been introduced. The current situation reflects the non-pharmaceutical interventions adopted by Malawi at the beginning of the outbreak. Enhanced shielding refers to reducing contact rates of people aged ≥ 60 years. Lockdown is the adoption of stringent social distancing policies. Further details are presented in Table 1. The trigger date is shown with a vertical grey dashed line. The red horizontal dashed line shows the capacity of the health system for non-intensive care (C). ICU capacity comprises 25 ICU beds and 16 mechanical ventilators.[33]

For peer review only

Figure 3. Impact of face covering on the total number of deaths per 1,000 population projected to occur over 250 days. The isoclines (green lines) represent the estimated *Rt* given the percentage efficacy and percentage of proper use (adherence). The impact of face covering (and enhanced shielding [B]) is assumed to be incremental to the current interventions in place.

Figure 4. Projected total numbers of deaths per 1,000 people over 250 days with increases in hospital capacity (and oxygen) and a novel therapeutic agent.

References

1. World Health Organization. Coronavirus disease (COVID-19): Situation Report - 209. Geneva, Switzerland, 2020.
2. World Health Organization A. WHO Africa Dashboard. 2020.
3. Cowling BJ, Ali ST, Ng TW, et al. Impact assessment of non-pharmaceutical interventions against COVID-19 and influenza in Hong Kong: an observational study. *MedRxiv* 2020.
4. Ferguson N, Laydon D, Nedjati Gilani G, et al. Report 9: Impact of non-pharmaceutical interventions (NPIs) to reduce COVID19 mortality and healthcare demand. 2020.
5. Flaxman S, Mishra S, Gandy A, et al. Estimating the effects of non-pharmaceutical interventions on COVID-19 in Europe. *Nature* 2020.
6. Kucharski AJ, Klepac P, Conlan A, et al. Effectiveness of isolation, testing, contact tracing and physical distancing on reducing transmission of SARS-CoV-2 in different settings. *medRxiv* 2020.
7. Davies NG, Kucharski AJ, Eggo RM, et al. Effects of non-pharmaceutical interventions on COVID-19 cases, deaths, and demand for hospital services in the UK: a modelling study. *The Lancet Public Health* 2020.
8. Teachout M, Zipfel C. The economic impact of COVID-19 lockdowns in sub Saharan Africa: International Growth Centre, 2020.
9. World Health Organization. Advice on the use of masks in the context of COVID-19. Geneva, Switzerland, 2020.
10. The Royal Society. Face masks and coverings for the general public: Behavioural knowledge, effectiveness of cloth coverings and public messaging. London, UK, 2020.
11. National Statistical Office, ICF. Malawi Demographic and Health Survey 2015–2016. 2017.
12. Sonenthal PD, Masiye J, Kasomekera N, et al. COVID-19 preparedness in Malawi: a national facility-based critical care assessment. *The Lancet Global Health* 2020.
13. UNICEF. Malawi: COVID-19 Situation Report No. 6. Lilongwe, Malawi, 2020.
14. Patel BK, Kress JP, Hall JB. Alternatives to Invasive Ventilation in the COVID-19 Pandemic. *JAMA* 2020; **324**(1): 43-4.
15. Horby P, Lim WS, Emberson J, et al. Effect of Dexamethasone in Hospitalized Patients with COVID-19: Preliminary Report. *medRxiv* 2020.
16. Wang Y, Zhang D, Du G, et al. Remdesivir in adults with severe COVID-19: a randomised, double-blind, placebo-controlled, multicentre trial. *The Lancet* 2020.
17. Banerjee A, Pasea L, Harris S, et al. Estimating excess 1-year mortality associated with the COVID-19 pandemic according to underlying conditions and age: a population-based cohort study. *The Lancet* 2020; **395**(10238): 1715-25.
18. Office for National Statistics. Deaths registered weekly in England and Wales, provisional: week ending 5 June 2020, 2020.
19. Verity R, Okell LC, Dorigatti I, et al. Estimates of the severity of coronavirus disease 2019: a model-based analysis. *The Lancet Infectious Diseases* 2020.
20. Davies MA. HIV and risk of COVID-19 death: a population cohort study from the Western Cape Province, South Africa. *medRxiv* 2020.

For peer review only

21. Palaiodimos L, Kokkinidis DG, Li W, et al. Severe obesity, increasing age and male sex are independently associated with worse in-hospital outcomes, and higher in-hospital mortality, in a cohort of patients with COVID-19 in the Bronx, New York. *Metabolism* 2020; **108**: 154262.
22. Ssentongo P, Ssentongo AE, Heilbrunn ES, Chinchilli VM. The association of cardiovascular disease and other pre-existing comorbidities with COVID-19 mortality: A systematic review and meta-analysis. *medRxiv* 2020.
23. Williamson E, Walker AJ, Bhaskaran KJ, et al. OpenSAFELY: factors associated with COVID-19-related hospital death in the linked electronic health records of 17 million adult NHS patients. *medRxiv* 2020.
24. Walker PGT, Whittaker C, Watson OJ, et al. The impact of COVID-19 and strategies for mitigation and suppression in low- and middle-income countries. *Science* 2020: eabc0035.
25. Melegaro A, Del Fava E, Poletti P, et al. Social contact structures and time use patterns in the Manicaland Province of Zimbabwe. *PloS one* 2017; **12**(1): e0170459.
26. Chibwana MG, Jere KC, Kamng'ona R, et al. High SARS-CoV-2 seroprevalence in Health Care Workers but relatively low numbers of deaths in urban Malawi. *medRxiv* 2020.
27. MRC Centre for Global Infectious Disease Analysis ICL. Situation Report for COVID-19: Africa, 2020-08-17. London, UK: Imperial College London; 2020.
28. Meyerowitz-Katz G, Merone L. A systematic review and meta-analysis of published research data on COVID-19 infection-fatality rates. *medRxiv* 2020.
29. Bell D, Hansen KS, Kiragga AN, Kambugu A, Kissa J, Mbonye AK. Predicting the Impact of COVID-19 and the Potential Impact of the Public Health Response on Disease Burden in Uganda. *Am J Trop Med Hyg* 2020.
30. Clark A, Jit M, Warren-Gash C, et al. Global, regional, and national estimates of the population at increased risk of severe COVID-19 due to underlying health conditions in 2020: a modelling study. *The Lancet Global Health* 2020.
31. Diop BZ, Ngom M, Biyong CP, Biyong JNP. The relatively young and rural population may limit the spread and severity of Covid-19 in Africa: a modelling study. *BMJ global health* 2020; **5**(5): e002699.
32. Kraemer MU, Yang C-H, Gutierrez B, et al. The effect of human mobility and control measures on the COVID-19 epidemic in China. *Science* 2020; **368**(6490): 493-7.
33. Nachega JB, Grimwood A, Mahomed H, et al. From Easing Lockdowns to Scaling-Up Community-Based COVID-19 Screening, Testing, and Contact Tracing in Africa – Shared Approaches, Innovations, and Challenges to Minimize Morbidity and Mortality. *Clinical Infectious Diseases* 2020.
34. World Bank. Databank. The World Bank Group; 2020.
35. Public Health England. Outbreaks in care homes in England, 2020.
36. Restrepo AMH, Moorthy V, Preziosi M-P. Public health emergency SOLIDARITY trial of treatments for COVID-19 infection in hospitalized patients. *ISRCTN* 2020.
37. Hellewell J, Abbott S, Gimma A, et al. Feasibility of controlling COVID-19 outbreaks by isolation of cases and contacts. *The Lancet Global Health* 2020.
38. Abdool Karim SS. The South African Response to the Pandemic. *New England Journal of Medicine* 2020.
39. Shapiro AE, Variava E, Rakgokong MH, et al. Community-based targeted case finding for tuberculosis and HIV in household contacts of patients with tuberculosis in South Africa. *American journal of respiratory and critical care medicine* 2012; **185**(10): 1110-6.
40. Reddy KP, Shebl FM, Foote JHA, et al. Cost-effectiveness of public health strategies for COVID-19 epidemic control in South Africa. *medRxiv* 2020.
41. Hogan AB, Jewell B, Sherrard-Smith E, et al. The potential impact of the COVID-19 epidemic on HIV, TB and Malaria in low-and middle-income countries. *Lancet Global Health* 2020; **Accepted: in press**.

For peer review only

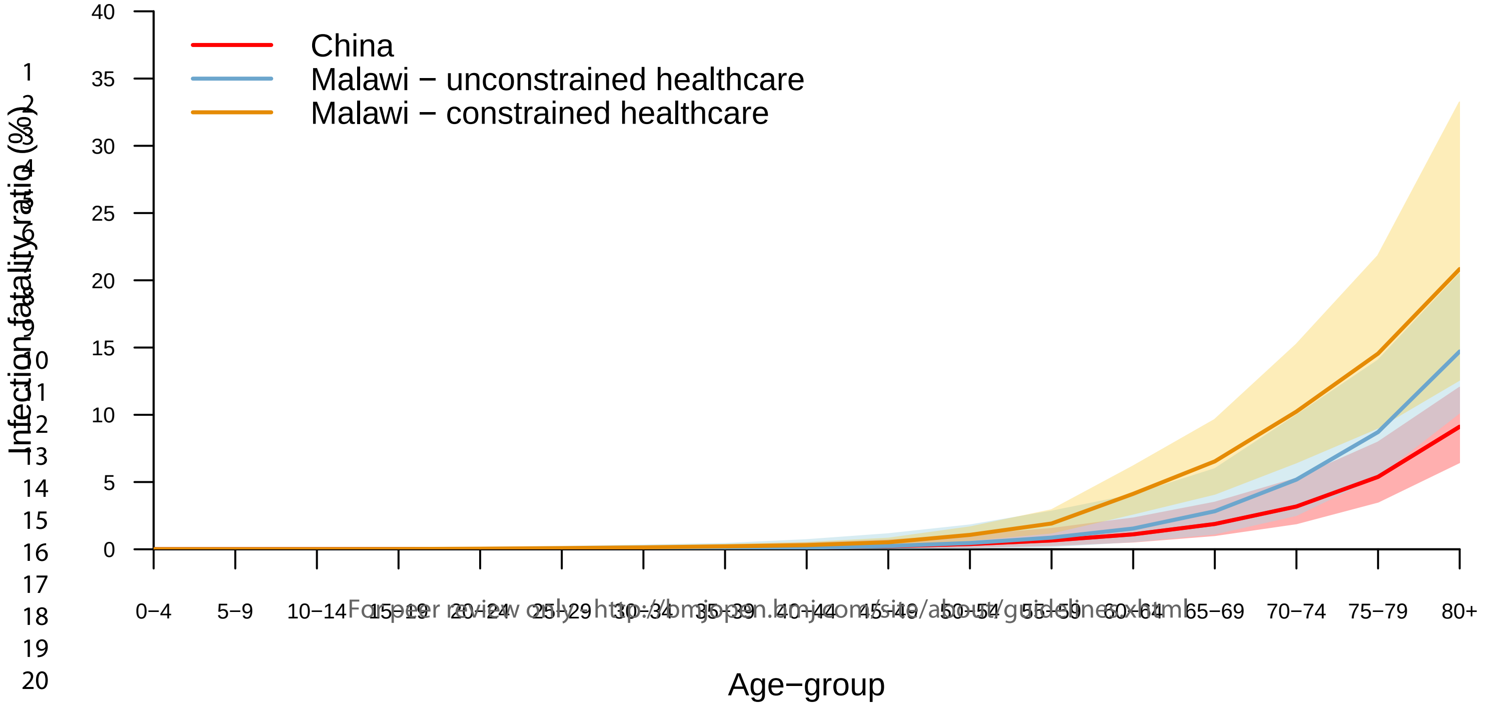

For peer review only - http://bmjopen.bmj.com/site/about/guidelines.xhtml

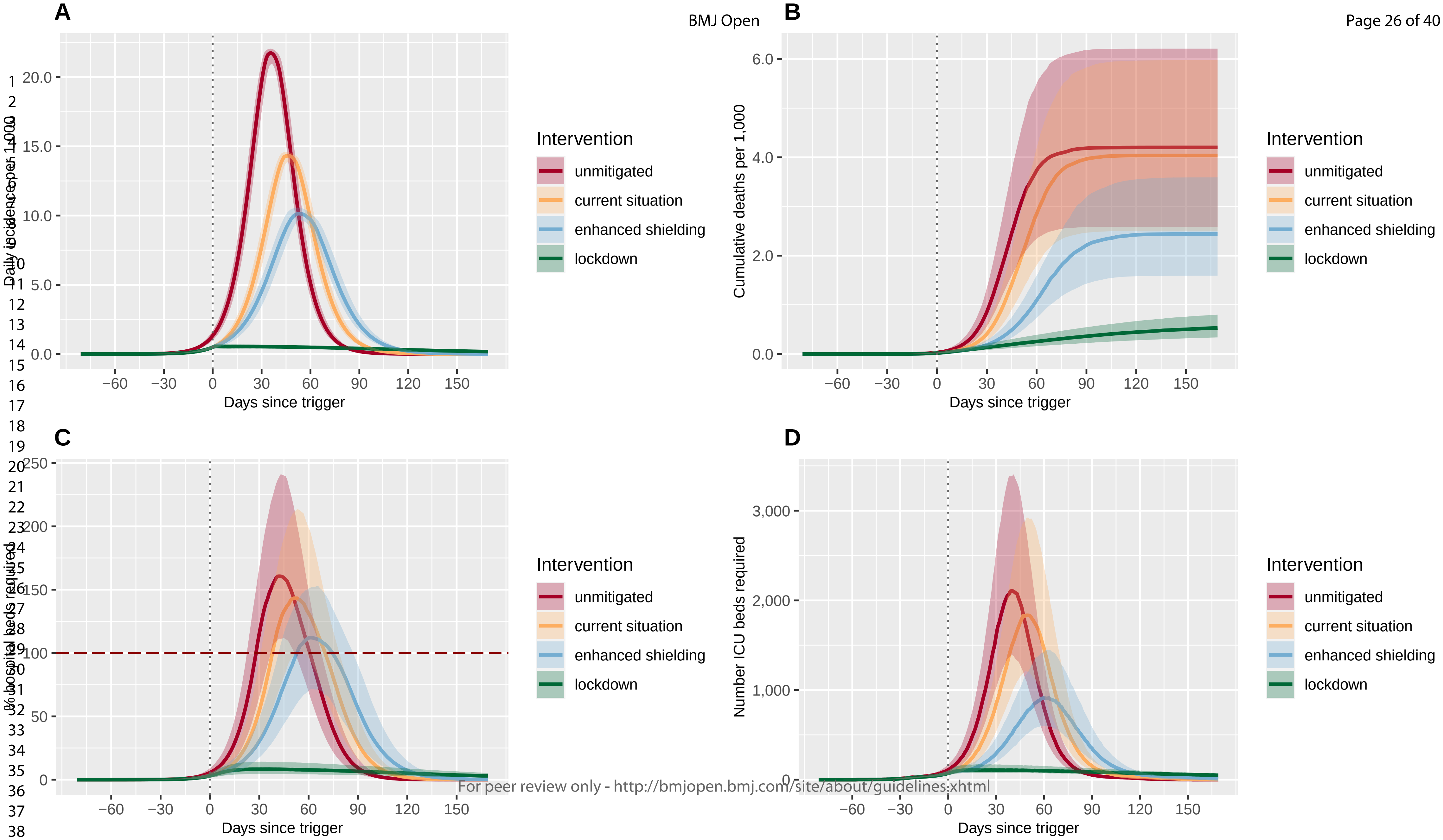

For peer review only - http://bmjopen.bmj.com/site/about/guidelines.xhtml

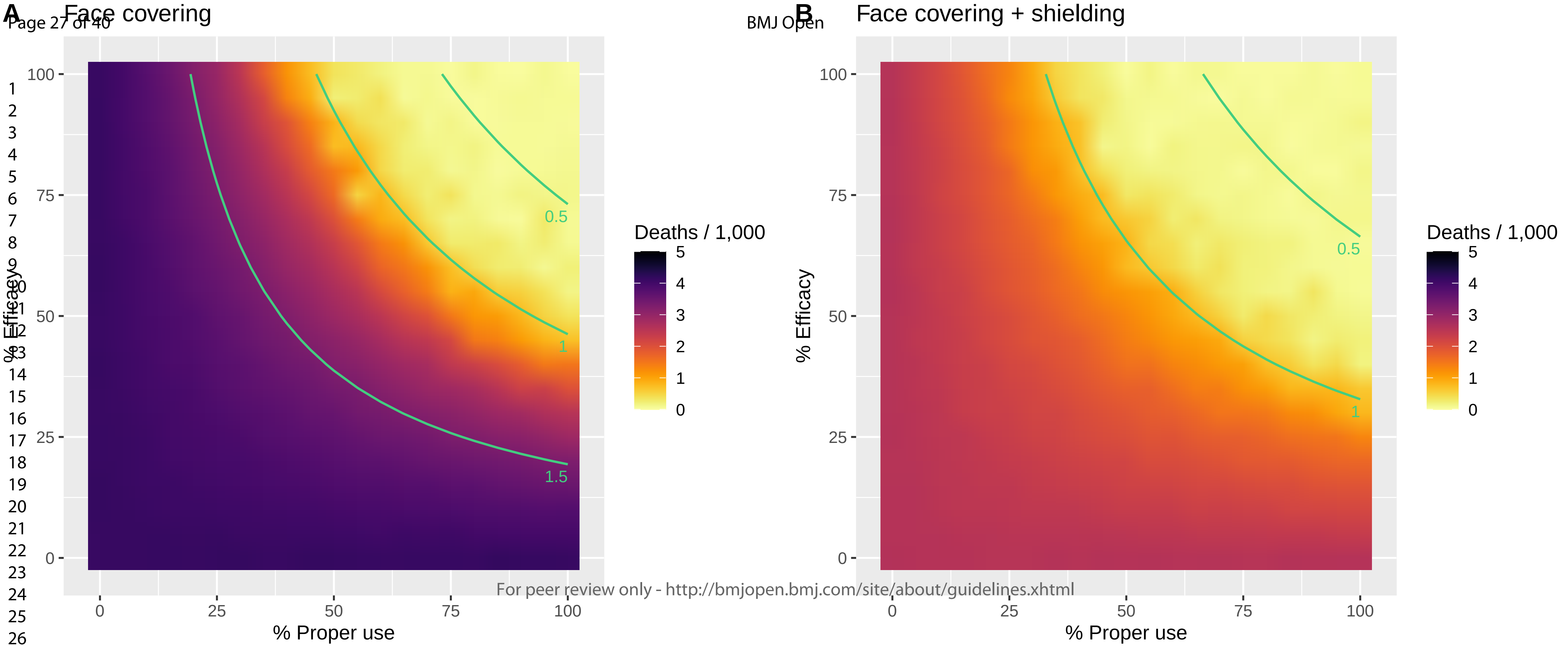
A
Face covering
B
Face covering + shielding
% Efficacy
% Proper use
0
25
50
75
100
0.5
1
1.5
Deaths / 1,000
5
4
3
2
1
0

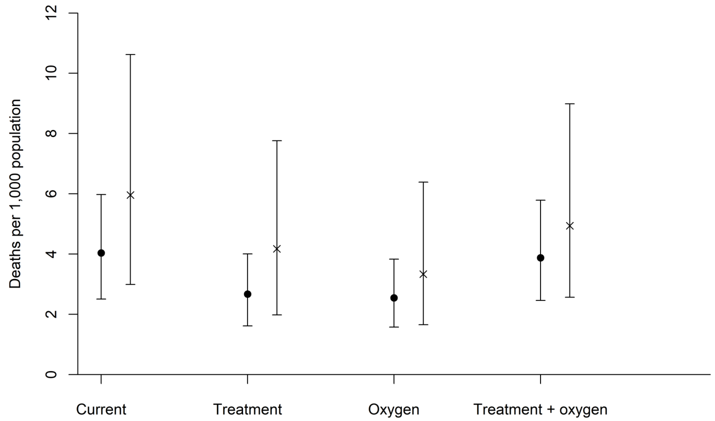

Intervention	Notes
Current	Assumes current interventions remain in place
Treatment	Current interventions in place plus availability of a therapeutic agent for all severe / critical cases (irrespective of hospital bed availability)
Oxygen	Current interventions in place plus 50% scale-up in hospital beds and oxygen availability
Treatment + oxygen	Current interventions in place plus 50% scale-up in hospital beds and oxygen availability and availability of a therapeutic agent for all severe / critical cases (irrespective of hospital bed availability)

Supplementary Information

Estimates of disease severity

Following Walker *et al*., we distinguish three levels of disease severity for COVID-19: (i) those that do not require hospitalisation; (ii) those that do require hospitalisation but not intensive care; (iii) those that require intensive care (ICU). Two parameters govern the proportions in each category: the proportion of infected cases requiring hospitalisation (*p_severe*) and the proportion of hospitalised cases requiring ICU (*p_critical*).

Uncertainty in these parameters is captured using prior information reported from high-income countries and generating sets of probabilities conditional on the resulting IFR falling within the bounds described in the methods. To do this, we sampled from a range of possible values for each parameter (detailed in Supplementary Table 5) based on values observed in high-income settings, keeping the age-distribution consistent with that reported in Verity *et al* and Walker *et al*.[1, 2] As we have based the calculation of IFR so far on data from China, we assumed that all persons that needed hospitalisation or intensive care received it, and that the proportion of those requiring intensive care that die, *m*, is 50%. This is a generalised assumption which incorporates both the availability of treatment and the mortality rates across both the untreated and treated critical cases. We assume no mortality in severe cases in this instance, although this assumption is changed in the simulation model. Combining the assumptions together, gives an overall IFR across the 17 age-groups as follows:

$$IFR_h = \sum_{a=1}^{17} (p\_severe_{h,a} * p\_critical_{h,a} * m) * N_a$$

Parameter sets for *p_severe* and *p_critical* were accepted if the resulting overall IFR fell within the 95% uncertainty interval of the adjusted IFR for Malawi and the process was repeated until 1000 accepted parameter sets were generated. The resulting parameter sets were used as inputs to the epidemic model.

For peer review only

Supplementary Table 1. Prevalence data used for the calculations of disease severity and infection fatality ratios.

Data	Source	Notes
TB	WHO TB Report [3]	The estimated incidence of active TB in 2018 is used, which is the latest year for which data were available: number of active cases divided by population size for every age-group and by sex
HIV	UNAIDS Country Projections 2019 [4]<br><br>IHME [5]	For Malawi: includes all PLHIV, irrespective of treatment status<br><br>For China: includes all PLHIV, irrespective of treatment status
Malaria	IHME [5]	For Malawi only. Prevalence of clinical malaria episodes in 2017 was used, which is the latest year for which data were available
COPD	IHME [5]	For both countries
CVD	IHME [5]	For both countries<br>Included as part of the CVD comorbidities
CKD	IHME [5]	For both countries<br>Included as part of the CVD comorbidities
Diabetes	Price *et al* [6]<br><br>IHME [5]	Included as part of the CVD comorbidities<br>Price et al 2018 for Malawi estimates and IHME 2020 for China estimates
Hypertension	Price *et al* [6]	Included as part of the CVD comorbidities<br>Price et al 2018 for Malawi estimates. Hypertension is included in the CVD prevalence estimates for the China
Obesity	Global Obesity Observatory [7]	Included as part of the CVD comorbidities<br>For both countries
Malnutrition	IHME [5]	For both countries
Population size	UNdata [8]	For both countries

All disease prevalence estimates are age- and sex-stratified.

For peer review only

Supplementary Table 2. Parameters used in the ICL Global COVID-19 Model

Parameter	Value	Notes / source
R_0	2	range 1.5-3.0
Hospital bed capacity	1.3 beds per 1,000 population	Equates to 24,869 hospital beds. Source: World Bank [9]
ICU bed capacity	25	
Mean latent period	4.6 days	Estimated at 5.1 days. The last 0.5 days are incorporated in the infectious periods to capture pre-symptomatic infectivity
Mean duration of mild infection	2.1 days	Incorporates 0.5 days of infectiousness prior to symptoms. In combination with mean duration of severe illness this gives a mean serial interval of 6.75 days
Mean duration of severe infection prior to hospitalisation	4.5 days	Mean onset-to-admission of 4 days based on unpublished analysis of data from the ICNARC study. Includes 0.5 days of infectiousness prior to symptom onset
Mean duration of hospitalisation for non-critical cases if survive	9.5 days	Based on unpublished analysis of data from the ICNARC study
Mean duration of hospitalisation for non-critical cases if die	7.6 days	Based on unpublished analysis of data from the ICNARC study
Mean duration in ICU if survive	11.3 days	Based on data from the ICNARC study adjusted for censoring
Mean duration in ICU if die	10.1 days	Based on data from the ICNARC study adjusted for censoring.
Mean duration in recovery after ICU	3.4 days	Based on unpublished analysis of data from the ICNARC study
Relative risk of mortality for patients with HIV	2.75	95% CI 2.09 – 3.61 [10]
Relative risk of mortality for patients with TB	2.58	95% CI 1.53 – 4.37 [10]
Relative risk of mortality for patients with malaria	1.5	Assumption, range 1-3
Relative risk of mortality for patients with cardiovascular disease	2.35	95% CI 1.44 – 3.84 [11]
Relative risk of mortality for patients with COPD	1.76	95% CI 0.92 – 3.36 [11]
Relative risk of mortality for patients with obesity	1	No additional risk once controlling for other comorbidities [12]
Relative risk of mortality for patients with malnutrition	1	Assumption, range 1-3

For peer review only

Supplementary Table 3. Age-dependent mortality rates

	**Severe cases**		**Critical cases**	
Age-group	Probability death with treatment	Probability death no treatment	Probability death with treatment	Probability death no treatment
0-4	0.013	0.026	0.5	0.95
5-9	0.013	0.026	0.5	0.95
10-14	0.013	0.026	0.5	0.95
15-19	0.013	0.026	0.5	0.95
20-24	0.013	0.026	0.5	0.95
25-29	0.013	0.026	0.5	0.95
30-34	0.013	0.026	0.5	0.95
35-39	0.013	0.026	0.5	0.95
40-44	0.015	0.026	0.5	0.95
45-49	0.019	0.026	0.5	0.95
50-54	0.027	0.026	0.5	0.95
55-59	0.042	0.084	0.5	0.95
60-64	0.069	0.138	0.5	0.95
65-69	0.105	0.210	0.5	0.95
70-74	0.149	0.298	0.5	0.95
75-79	0.203	0.406	0.5	0.95
80+	0.580	1.000	0.5	0.95

The probability of death in severe cases not receiving treatment has been changed from the default values in the ICL Global Model. Here we assume mortality rates for untreated severe cases are double those of the treated cases. Values are derived using data from the ICNARC study in the UK.[13]

For peer review only

Supplementary Table 4. Social contact matrix between age-groups in Zimbabwe

Age-group	**0-4**	**5-9**	**10-14**	**15-19**	**20-24**	**25-29**	**30-34**	**35-39**	**40-44**	**45-49**	**50-54**	**55-59**	**60-64**	**65-69**	**70-74**	**75+**
**0-4**	1.006	1.030	0.883	0.732	0.647	0.712	0.645	0.463	0.289	0.214	0.211	0.215	0.179	0.121	0.075	0.048
**5-9**	0.807	2.863	2.668	0.968	0.423	0.449	0.468	0.416	0.289	0.212	0.182	0.186	0.177	0.140	0.093	0.066
**10-14**	0.659	2.544	3.766	1.865	0.580	0.413	0.452	0.536	0.375	0.251	0.207	0.208	0.193	0.177	0.136	0.096
**15-19**	0.617	1.041	2.104	2.548	1.070	0.575	0.536	0.645	0.441	0.297	0.267	0.264	0.229	0.225	0.182	0.112
**20-24**	0.947	0.790	1.135	1.858	1.363	0.969	0.752	0.723	0.474	0.363	0.333	0.310	0.281	0.254	0.199	0.123
**25-29**	1.091	0.878	0.848	1.046	1.014	1.131	0.885	0.707	0.425	0.330	0.297	0.299	0.271	0.225	0.153	0.101
**30-34**	1.268	1.175	1.191	1.252	1.010	1.136	1.119	1.032	0.593	0.405	0.356	0.364	0.341	0.249	0.164	0.102
**35-39**	0.976	1.119	1.512	1.612	1.041	0.972	1.105	1.221	0.756	0.485	0.420	0.409	0.358	0.276	0.177	0.105
**40-44**	0.960	1.225	1.668	1.738	1.076	0.923	1.001	1.192	0.863	0.629	0.488	0.434	0.378	0.311	0.207	0.117
**45-49**	0.796	1.007	1.248	1.312	0.922	0.801	0.766	0.856	0.704	0.549	0.446	0.383	0.352	0.298	0.215	0.120
**50-54**	0.844	0.928	1.106	1.266	0.909	0.774	0.722	0.796	0.587	0.479	0.417	0.407	0.397	0.331	0.242	0.159
**55-59**	0.902	0.993	1.163	1.312	0.888	0.818	0.776	0.812	0.547	0.431	0.426	0.483	0.484	0.382	0.281	0.210
**60-64**	0.823	1.041	1.191	1.250	0.882	0.815	0.797	0.781	0.524	0.435	0.457	0.532	0.567	0.449	0.347	0.259
**65-69**	0.695	1.026	1.357	1.534	0.995	0.841	0.725	0.751	0.537	0.459	0.475	0.524	0.560	0.471	0.372	0.333
**70-74**	0.592	0.943	1.442	1.712	1.080	0.791	0.664	0.668	0.495	0.458	0.481	0.532	0.599	0.515	0.455	0.383
**75+**	0.557	0.974	1.485	1.530	0.967	0.763	0.597	0.577	0.406	0.373	0.458	0.579	0.648	0.669	0.556	0.400

Values are estimated number of contacts between persons in each age-group per day. The contact matrix is adjusted to give age-specific contact rates.[14]

Supplementary Table 4. List of the interventions under consideration along with their implementation in the model.

**Strategy**	**Implementation**
Unmitigated epidemic baseline	Includes only isolation of hospitalised cases
**Current situation:**<br><br>Implemented from 18th April 2020	Consider these strategies as a bundle equating to a combined reduction in *Rt* of 7% and a reduction in contact rates for the whole population plus additional reductions for employed people and school-aged children as detailed below.<br><br>Dates and details of individual non-pharmaceutical interventions are reported by OxCGRT[15]
Social distancing recommended	Reduce contact rates for the whole population by 24% (maximum reduction in population movement reported by Google Mobility Reports)[16]#
Workplace closures	Reduce contact rates of those employed in offices and with access to internet at home (6·4% of the population aged 15-49) by 50% (this value will include social distancing so additional reductions of 26% are applied to the eligible population)[17]
School closures	Reduce contact rates between children aged 5-15 years by 50% (this value will include social distancing so additional reductions of 26% are applied to the eligible population)
Public events cancelled	Relative reduction in *Rt* 4% range 1-17%. We take the mid-point of the estimated relative reduction in *Rt* for each of these individual strategies[18]
Self-quarantine for returning individuals	Relative reduction in *Rt* 3% range 0-15%. We take the mid-point of the estimated relative reduction in *Rt* for each of these individual strategies[18]
Restriction of gatherings to 10	Assume no impact above that of cancelling public events
International travel controls	Assume no new infections imported from other countries
**Enhanced Shielding of the Elderly:**<br><br>Current situation, plus:<br><br>Shielding the elderly	Reduce contact rates by 40% for populations aged ≥60 years in addition to the reduction in *Rt* and contact rates above.<br><br>It is implemented after a trigger is reached$

For peer review only

**Lockdown:**<br><br>Current situation, plus:<br><br>Shelter in place<br><br>Restrict movements to essential activities such as the provision of essential goods and services<br><br>Enforce social distancing in excepted businesses<br><br>Prohibit public transportation<br><br>Prohibit all gatherings outside household	Consider that this bundle equates to a sustained reduction in *Rt* to 1. Estimated Rt <1 has been reported in low-income settings following lockdowns, we assume *Rt* would not fall below 1 due to continued household transmission and potential non-compliance.[19] The reductions in contact rates listed under "current situation" are also assumed to apply in this scenario.<br><br>It is implemented after a trigger is reached[$]

[#]Data are from Zambia, data from Malawi are not available. Note Zambia rural population is 56·5% compared with Malawi 83·1%.

[$]The trigger date for interventions to be applied was 1·0 COVID-19 deaths per 100,000 population per week

For peer review only

Supplementary Table 6. Estimates of disease severity for the Malawian population

Age-group (years)	Proportion cases requiring hospitalisation		Proportion hospitalised cases requiring ICU	
	Credible range	Median accepted values (2.5th and 97.5th quantiles) *	Credible range	Median accepted values (2.5th and 97.5th quantiles) *
0-4	0.0002-0.005	0.0013 (0.0003 - 0.0028)	0.01-0.25	0.0624 (0.016 - 0.1434)
5-9	0.0002-0.005	0.0013 (0.0003 - 0.0028)	0.01-0.25	0.0624 (0.016 - 0.1434)
10-14	0.0002-0.005	0.0013 (0.0003 - 0.0028)	0.01-0.25	0.0624 (0.016 - 0.1434)
15-19	0.0004-0.01	0.0026 (0.0007 - 0.0057)	0.01-0.25	0.0624 (0.016 - 0.1434)
20-24	0.001-0.025	0.0064 (0.0017 - 0.0142)	0.01-0.25	0.0624 (0.016 - 0.1434)
25-29	0.002-0.05	0.0128 (0.0034 - 0.0284)	0.01-0.25	0.0624 (0.016 - 0.1434)
30-34	0.0032-0.08	0.0205 (0.0054 - 0.0454)	0.01-0.25	0.0624 (0.016 - 0.1434)
35-39	0.0046-0.115	0.0295 (0.0077 - 0.0653)	0.0106-0.265	0.0662 (0.0169 - 0.152)
40-44	0.0058-0.145	0.0372 (0.0097 - 0.0823)	0.012-0.3	0.0749 (0.0192 - 0.1721)
45-49	0.0078-0.195	0.05 (0.0131 - 0.1107)	0.015-0.375	0.0937 (0.024 - 0.2151)
50-54	0.0116-0.29	0.0744 (0.0195 - 0.1646)	0.0208-0.52	0.1299 (0.0332 - 0.2983)
55-59	0.0144-0.36	0.0923 (0.0242 - 0.2044)	0.0298-0.745	0.1861 (0.0476 - 0.4274)
60-64	0.0204-0.51	0.1308 (0.0342 - 0.2895)	0.0448-1.12	0.2797 (0.0716 - 0.6425)
65-69	0.0234-0.585	0.15 (0.0393 - 0.3321)	0.0614-1.535	0.3834 (0.0981 - 0.8806)
70-74	0.0292-0.73	0.1872 (0.049 - 0.4144)	0.0772-1.93	0.4821 (0.1233 - 1)
75-79	0.0354-0.885	0.2269 (0.0594 - 0.5024)	0.0922-2.305	0.5757 (0.1473 - 1)
80+	0.036-0.9	0.2308 (0.0604 - 0.5109)	0.1418-3.545	0.8854 (0.2265 - 1)

* distributions obtained through rejection sampling.

For peer review only

Supplementary Table 7. Simulation outputs under different assumptions of R_0.

		Baseline	Current situation	Enhanced shielding	Lockdown
$R_0 = 1.5$	Total cases / 1,000	553 (462-642)	319 (258-370)	163 (130-195)	40 (27-52)
	Number hospital beds required at peak	23300 (13,400-33,500)	16800 (9,500-27,300)	7600 (4,600-12,100)	1200 (700-2,300)
	Number ICU beds required at peak	1,000 (600-1,500)	700 (500-1,100)	200 (200-300)	100 (100-100)
	Total number of deaths	56900 (36,100-83,800)	39800 (25,600-60,100)	11500 (7,800-17,200)	5400 (3,200-8,400)
$R_0 = 2.0$	Total cases / 1,000	770 (666-875)	584 (507-662)	513 (436-595)	71 (58-85)
	Number hospital beds required at peak	40,000 (27,800-59,900)	35,600 (25,300-53,100)	27,900 (17,800-38,000)	2,100 (1,100-3,500)
	Number ICU beds required at peak	2,100 (1,400-3,400)	1,800 (1,200-2,900)	900 (600-1,400)	100 (100-200)
	Total number of deaths	80,400 (49,500-118,800)	77,200 (48,000-114,300)	46,700 (30,400-68,700)	10,100 (6,500-15,300)
$R_0 = 3.0$	Total cases / 1,000	920 (815-1,026)	756 (669-844)	716 (627-808)	109 (93-125)
	Number hospital beds required at peak	61,700 (41,200-98,800)	57,600 (38,700-92,000)	49,700 (34,200-75,400)	4,100 (2,400-6,900)
	Number ICU beds required at peak	3,800 (2,500-6,000)	3,500 (2,300-5,600)	2,300 (1,500-3,700)	200 (100-300)
	Total number of deaths	93,000 (57,700-136,400)	92,100 (57,200-134,800)	72,000 (44,900-105,500)	15,800 (10,400-23,600)

All values are medians of 1000 simulations using the sampled parameter sets. Numbers of hospital beds, ICU beds and deaths are rounded to the nearest 100. The trigger day for interventions (1.0 death per 100,000 population per week) is day 145, 82, and 52 for R_0=1.5, 2.0 and 3.0 respectively.

For peer review only

Supplementary Table 8. Projected cumulative number of deaths with increases in hospital capacity.

	**Low mortality**	**High mortality**
**Intervention**	Total deaths per 1,000 population	Total deaths per 1,000 population
**Current capacity**	4·04<br>(2·51-5·98)	5·96<br>(2·99-10·62)
**Increase capacity by 25%**	3·95<br>(2·48-5·89)	5·41<br>(2·75-9·75)
**Increase capacity by 50%**	3·89<br>(2·47-5·79)	4·94<br>(2·58-8·98)
**Increase capacity by 75%**	3·82<br>(2·47-5·70)	4·66<br>(2·51-8·43)
**Increase capacity by 100%**	3·79<br>(2·45-5·61)	4·41<br>(2·48-7·87)

Low and high mortality refer to the assumptions on mortality rates of untreated severe cases. Low mortality rates are those used in the main analysis (detailed in Supplementary Table 3) and high mortality applies a mortality rate of 0·6 across all age-groups.

For peer review only

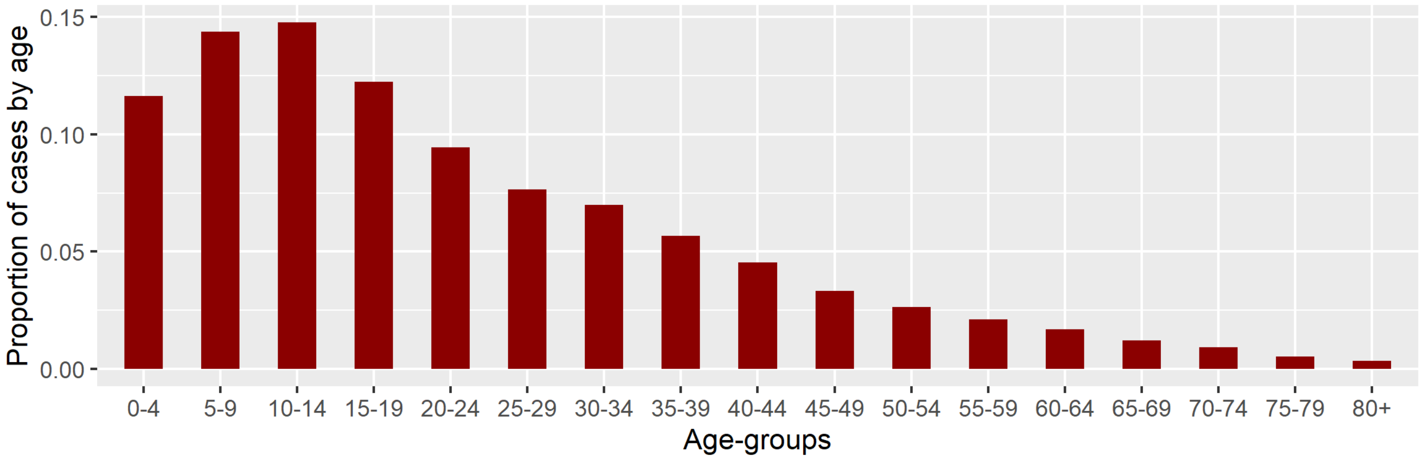

Supplementary Figure 1. Age distribution of infected cases (asymptomatic and symptomatic) at the peak of the projected unmitigated epidemic.

For peer review only

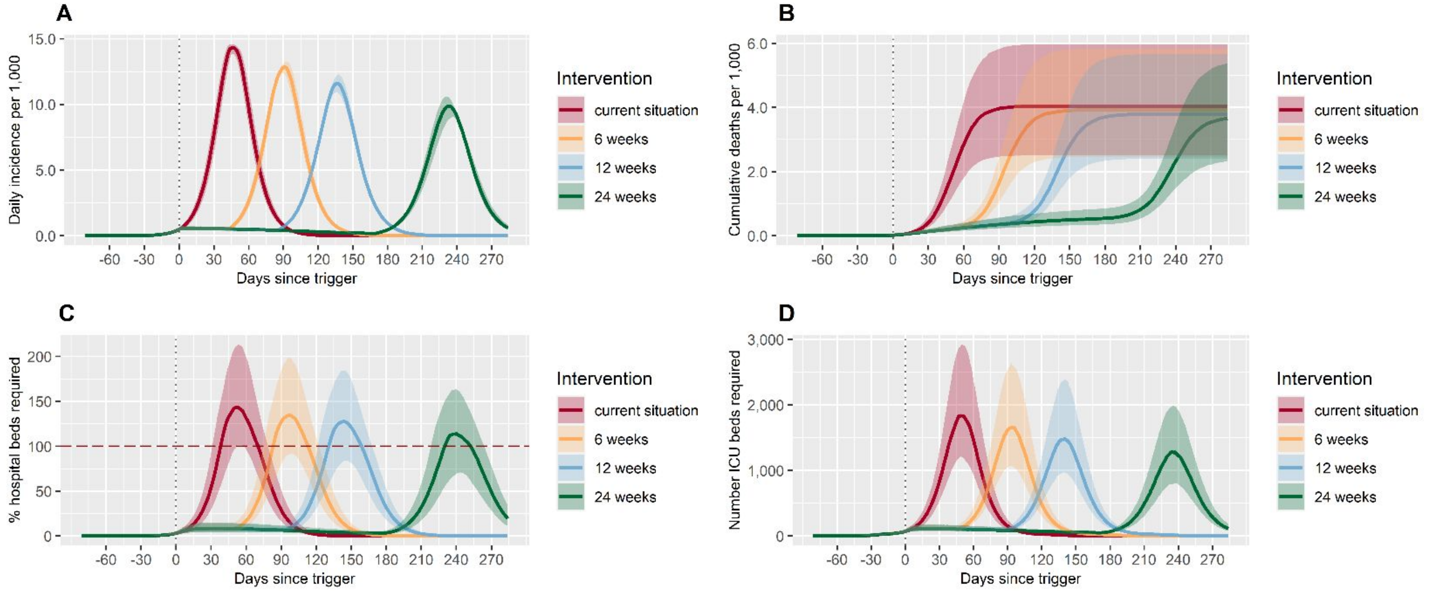

Supplementary Figure 2. Impact of changing the duration of lockdown compared with the current situation on the daily incidence per 1,000 population (A), the cumulative deaths per 1,000 population (B), the percentage of hospital beds that are required (C) and the number of ICU beds that are required (D). The current situation reflects the non-pharmaceutical interventions adopted by Malawi at the beginning of the outbreak. Each intervention represents the implementation of lockdown over 6, 12 or 24 weeks. The trigger date (1 death per 1,000 population per week) is shown with a vertical grey dashed line. The red horizontal dashed line shows the capacity of the health system for non-intensive care (C).

peer review only

References

1. Verity, R., et al., *Estimates of the severity of coronavirus disease 2019: a model-based analysis.* The Lancet Infectious Diseases, 2020.
2. Walker, P., et al., *Report 12: The global impact of COVID-19 and strategies for mitigation and suppression.* 2020.
3. World Health Organization, *WHO TB burden estimates*. 2020: Geneva, Switzerland.
4. UNAIDS, *AIDSInfo*. 2020: Geneva, Switzerland.
5. Global Burden of Disease Collaborative Network, *Global Burden of Disease Study 2017 (GBD 2017) Results*. 2018, Institute for Health Metrics and Evaluation (IHME): Seattle, USA.
6. Price, A.J., et al., *Prevalence of obesity, hypertension, and diabetes, and cascade of care in sub-Saharan Africa: a cross-sectional, population-based study in rural and urban Malawi.* The lancet Diabetes & endocrinology, 2018. **6**(3): p. 208-222.
7. World Obesity Federation, *Global Obesity Observatory*. 2020.
8. United Nations Statistics Division, *Population by age, sex and urban/rural residence*. 2020: New York, USA.
9. The World Bank, *World Bank Open Data [Hospital beds per 1,000 people]*. 2020.
10. Davies, M.-A. and Western Cape Department of Health. *Western Cape: COVID-19 and HIV / Tuberculosis*. 2020 10th June 2020]; Available from: https://storage.googleapis.com/stateless-bhekisisa-website/wordpress-uploads/2020/06/df4fcf94-covid_update_bhekisisa_wc_3.pdf.
11. Ssentongo, P., et al., *The association of cardiovascular disease and other pre-existing comorbidities with COVID-19 mortality: A systematic review and meta-analysis.* medRxiv, 2020.
12. Palaiodimos, L., et al., *Severe obesity, increasing age and male sex are independently associated with worse in-hospital outcomes, and higher in-hospital mortality, in a cohort of patients with COVID-19 in the Bronx, New York.* Metabolism, 2020. **108**: p. 154262.
13. Intensive Care National Audit & Research Centre *ICNARC report of COVID-19 in critical care*. 2020.
14. Melegaro, A., et al., *Social contact structures and time use patterns in the Manicaland Province of Zimbabwe.* PloS one, 2017. **12**(1).
15. Hale, T., et al., *Oxford COVID-19 Government Response Tracker, Blavatnik School of Government*. 2020: University of Oxford.
16. Google LLC, *Google COVID-19 Community Mobility Reports*. 2020.
17. National Statistical Office and ICF, *Malawi Demographic and Health Survey 2015–2016.* 2017.
18. Flaxman, S., et al., *Estimating the effects of non-pharmaceutical interventions on COVID-19 in Europe.* Nature, 2020.
19. MRC Centre for Global Infectious Disease Analysis, I.C.L., *Situation Report for COVID-19: Africa, 2020-08-17*. 2020, Imperial College London: London, UK.

For peer review only

BMJ Open

The potential impact of intervention strategies on COVID-19 transmission in Malawi: A mathematical modelling study

Journal:	*BMJ Open*
Manuscript ID	bmjopen-2020-045196.R1
Article Type:	Original research
Date Submitted by the Author:	24-Mar-2021

Complete List of Authors:	Mangal, Tara; Imperial College London, Infectious Disease Epidemiology<br>Whittaker, Charlie; Imperial College London, Infectious Disease Epidemiology<br>Nkhoma, Dominic; University of Malawi College of Medicine<br>Ng'ambi, Wingston; University of Malawi College of Medicine<br>Watson, Oliver; Imperial College London School of Public Health, Infectious Disease Epidemiology<br>Walker, Patrick; Imperial College London, Infectious Disease Epidemiology<br>Ghani, AC; Imperial College London, Infectious Disease Epidemiology<br>Revill, Paul; University of York, Centre for Health Economics<br>Colbourn, Timothy; UCL Institute for Global Health,<br>Phillips, Andrew; UCL, HIV Epidemiology and Biostatistics Group<br>Hallett, Timothy; Imperial College London, Infectious Disease Epidemiology<br>Mfutso-Bengo, Joseph; University of Malawi College of Medicine

**Primary Subject Heading**:	Epidemiology
Secondary Subject Heading:	Global health, Infectious diseases, Public health
Keywords:	Public health < INFECTIOUS DISEASES, COVID-19, EPIDEMIOLOGY, Infection control < INFECTIOUS DISEASES

SCHOLARONE™ Manuscripts

I, the Submitting Author has the right to grant and does grant on behalf of all authors of the Work (as defined in the below author licence), an exclusive licence and/or a non-exclusive licence for contributions from authors who are: i) UK Crown employees; ii) where BMJ has agreed a CC-BY licence shall apply, and/or iii) in accordance with the terms applicable for US Federal Government officers or employees acting as part of their official duties; on a worldwide, perpetual, irrevocable, royalty-free basis to BMJ Publishing Group Ltd ("BMJ") its licensees and where the relevant Journal is co-owned by BMJ to the co-owners of the Journal, to publish the Work in this journal and any other BMJ products and to exploit all rights, as set out in our licence.

The Submitting Author accepts and understands that any supply made under these terms is made by BMJ to the Submitting Author unless you are acting as an employee on behalf of your employer or a postgraduate student of an affiliated institution which is paying any applicable article publishing charge ("APC") for Open Access articles. Where the Submitting Author wishes to make the Work available on an Open Access basis (and intends to pay the relevant APC), the terms of reuse of such Open Access shall be governed by a Creative Commons licence – details of these licences and which Creative Commons licence will apply to this Work are set out in our licence referred to above.

Other than as permitted in any relevant BMJ Author's Self Archiving Policies, I confirm this Work has not been accepted for publication elsewhere, is not being considered for publication elsewhere and does not duplicate material already published. I confirm all authors consent to publication of this Work and authorise the granting of this licence.

For peer review only

Title: The potential impact of intervention strategies on COVID-19 transmission in Malawi: A mathematical modelling study

Authors: Tara D. Mangal[1] PhD, Charlie Whittaker[1], Dominic Nkhoma[2] PhD, Wingston Ng'ambi[2], Oliver J Watson[1], Patrick Walker[1] PhD, Azra Ghani[1] PhD, Paul Revill[3] MSc, Tim Colbourn[4] PhD, Andrew Phillips[4] PhD, Timothy B. Hallett[1] PhD, Joseph Mfutso-Bengo[2] PhD

Affiliations:

[1]Medical Research Council Centre for Global Infectious Disease Analysis & Abdul Latif Jameel Institute for Disease and Emergency Analytics, Imperial College London, London, UK

[2]Health Economics & Policy Unit, College of Medicine, University of Malawi, Malawi

[3]Centre for Health Economics, University of York, UK

[4]Institute for Global Health, University College London, UK

Address for correspondence: Tara Mangal, Department of Infectious Disease Epidemiology, School of Public Health, Faculty of Medicine, Imperial College London, Norfolk Place, London W2 1PG; email: t.mangal@imperial.ac.uk

Keywords: SARS-CoV-2, COVID-19, Malawi, non-pharmaceutical interventions, infection fatality rate, comorbidity, face covering, severity, mortality.

For peer review only

Abstract

Background

COVID-19 mitigation strategies have been challenging to implement in resource-limited settings due to the potential for widespread disruption to social and economic well-being. Here we predict the clinical severity of COVID-19 in Malawi, quantifying the potential impact of intervention strategies and increases in health system capacity.

Methods

The infection fatality ratios (IFR) were predicted by adjusting reported IFR for China accounting for demography, the current prevalence of comorbidities and health system capacity. These estimates were input into an age-structured deterministic model, which simulated the epidemic trajectory with non-pharmaceutical interventions and increases in health system capacity.

Findings

The predicted population-level IFR in Malawi, adjusted for age and comorbidity prevalence, is lower than estimated for China (0.26%, 95% uncertainty interval [UI] 0.12 – 0.69%, compared with 0.60%, 95% CI 0.4% – 1.3% in China), however the health system constraints increase the predicted IFR to 0.83%, 95% UI 0.49% – 1.39%. The interventions implemented in January 2021 could potentially avert 54,400 deaths (95% UI 26,900 – 97,300) over the course of the epidemic compared with an unmitigated outbreak. Enhanced shielding of people aged ≥ 60 years could avert a further 40,200 deaths (95% UI 25,300 – 69,700) and halve ICU admissions at the peak of the outbreak. A novel therapeutic agent, which reduces mortality by 0.65 and 0.8 for severe and critical cases respectively, in combination with increasing hospital capacity could reduce projected mortality to 2.5 deaths per 1,000 population (95% UI 1.9 – 3.6).

Conclusion

For peer review only

We find the interventions currently used in Malawi are unlikely to effectively prevent SARS-CoV-2 transmission but will have a significant impact on mortality. Increases in health system capacity and the introduction of novel therapeutics are likely to further reduce the projected numbers of deaths.

For peer review only

Strengths and limitations of this study

- This is the first study to date which combines country-specific infection fatality ratios (IFR) of COVID-19 for Malawi adjusted for comorbidity prevalence and with consideration to the prevailing health system constraints and the impact of these constraints on mortality rates.
- An age-structured deterministic model was used to characterise the spread of SARS-CoV-2 throughout Malawi using the Malawi-specific adjusted IFR.
- The impacts of non-pharmaceutical interventions, novel therapeutics and hospital capacity were analysed and the effects on incidence, mortality and hospital demand are presented.
- The IFR used as the baseline, from which we infer the adjusted IFR for Malawi, and some key parameters used in the simulation modelling rely on data from outside sub-Saharan Africa due to the limited numbers of cases there and may therefore not be directly transferable to Malawi.

For peer review only

Author's contributions

Literature search: TDM, TC, AP, TBH

Figures: TDM

Study design: TDM, CW, OJW, PW, AG, TC, AP, TBH, JM-B

Data collection: TDM, CW, DN, OJW, PW, AG, TBH, JM-B

Data analysis: TDM, TBH, AP, TC

Data interpretation: TDM, CW, DN, OJW, PW, AG, PR, TC, AP, TBH, JM-B

Writing: TDM, CW, DN, WN, OJW, PW, AG, PR, TC, AP, TBH, JM-B

INTRODUCTION

As of 15th March 2021, the novel coronavirus severe acute respiratory syndrome coronavirus 2 (SARS-CoV-2) had spread throughout every continent, with over 100 million cases and 2.5 million deaths reported worldwide.(1) Case numbers in the African continent continue to rise and until widespread deployment of an effective vaccine, there is a critical reliance on non-pharmaceutical interventions (NPI) to reduce transmission . These measures include isolation of suspected/confirmed cases, contact tracing, social distancing, travel restrictions, face covering, school and workplace closures and shielding of the most vulnerable.(2-5)

By the 1st January 2021, Malawi was experiencing a second wave of infections and additional restrictions were placed on the population. Schools and workplaces were closed in many districts, large gatherings and public events were banned, but a full lockdown has been prohibited due to concerns around the implications on vulnerable populations.(6) The impact of NPI on SARS-CoV-2 transmission in Malawi depends critically on the local context such as population behaviour (including uptake of and compliance with such measures), population movement and contact patterns (Malawi is over 80% rural and many rely on subsistence farming) and health system capacity.(7)

The impact of NPI can be summarised as a change in the effective reproduction number *Rt*, which represents the average number of secondary infections resulting from one infected case. Studies based in high-income countries have shown that strict interventions such as lockdown, where population movement is limited to essential travel and most public facilities and transport links are closed, have shown the most success in reducing transmission.(3, 4, 8-10) However major restrictions to working practices or public transport may have catastrophic implications in Sub-Saharan Africa, where many have limited financial capacity to withstand income shocks and no access to social protection programmes.(11) Face coverings have been recommended by WHO as one possible intervention which could reduce transmission of SARS-CoV-2 with minimal socioeconomic implications despite a lack of high-quality evidence.(12, 13) Nevertheless, many countries, (including, Canada, South Korea and the UK), have made face coverings mandatory in public spaces.

For peer review only

A key priority during this emerging pandemic, is estimating clinical severity and health system requirements. Current oxygen capacity in hospitals may not be sufficient to give supportive care to large numbers of severe COVID-19 cases; 65% of 34 hospital wards in Malawian hospitals recently assessed had (what was considered pre-COVID-19) adequate access to oxygen and priority is now being given to scaling up access and supplies urgently.(14, 15) Early studies suggest oxygen supplementation could reduce the need for mechanical ventilation and lower the risk of death.(16) Additionally, several therapeutic agents, such as tocilizumab and dexamethasone, have been effective in improving patient outcomes, although none so far have been widely tested in sub-Saharan Africa.(17, 18)

Infection fatality ratios (IFR), defined as the number of deaths divided by the number of infections are challenging to estimate, particularly in an emerging outbreak, due to the difficulties in identifying the true number of infected people (both asymptomatic and symptomatic). IFR are strongly dependent on age and the majority of deaths reported early in the epidemic were among those aged over 60 years.(19-21) There is also a growing body of evidence on the elevated risk of mortality with certain underlying comorbidities, such as cardiovascular disease (CVD), diabetes, chronic obstructive pulmonary disease (COPD) and infectious diseases, for example HIV, TB and malaria.(22-25) The majority of these data were reported from high-income settings, which have borne the highest burden of disease recorded so far. It is not yet clear how these risk factors will affect disease severity in countries like Malawi, which have a younger population overall, but a high prevalence of infectious diseases and untreated chronic conditions.

Objectives

Three objectives form the focus of this paper: 1) predict disease severity caused by SARS-CoV-2 in the Malawian population given its demographic structure, the prevalence of key comorbidities and health system capacity; 2) examine the potential impact of a range of NPI that have been or could be used in Malawi; 3) on that basis, investigate the potential extent to which increasing health system capacity and/or providing therapeutics could contribute to reducing deaths due to SARS-CoV-2 infection.

For peer review only

METHODS

We present the methods in three sections that relate to each of our aims.

1. Predictions of infection fatality ratios in Malawi

Our approach utilises data on age-specific IFR from China (one of the few studies which applies demography-adjusted under-ascertainment corrections) and then makes adjustments based on the demography and relative burdens of diseases relevant to COVID-19 risk between China and Malawi, making assumptions about the extent to which each disease affects IFR and the extent and impact of healthcare available.(21) First, we predict IFR by age under the assumption of similar availability and impact of healthcare. We then use these predicted IFR and adjust for the potential impact of a constrained healthcare system in Malawi, making assumptions on the effect of treatment on mortality rates of severe and critical cases. The predicted IFR therefore represent pooled estimates of those receiving and not receiving care.

i) Predicted IFR with an unconstrained health system

The prevalence of HIV (virally suppressed and unsuppressed), active TB, clinical malaria, CVD, COPD, hypertension, diabetes (type I and II), obesity (defined as BMI ≥28 kg/m^2 according to Chinese criteria and ≥30 kg/m^2 using Malawian criteria) and malnutrition were extracted for Chinese and Malawian populations (Supplementary Figure 1, Supplementary Table 1 for data sources). We created a unified risk factor for "metabolic syndrome", defined as the presence of at least one of the following conditions which tend to be clustered within individuals: CVD, hypertension, obesity and diabetes. The plausible range for the risks of mortality due to metabolic syndrome were taken as the outer bounds of the relative risks reported for each of the pooled conditions. Given the considerable uncertainty in these estimates along with likely differences across settings, we sample from a wide range of relative risk values for each comorbidity (Supplementary Table 2).

The baseline age-distributed IFR were derived from those published by Verity *et al* for cases reported in mainland China, using linear interpolation on the log scale to derive values in five-year age-groups from the 10-year age-bands reported.(21) Adjusted IFR by age for Malawi were computed as follows:

For peer review only

1) Lognormal distributions were derived for each of the age-distributed IFR from China such that the mean matched the mean IFR and 95% of the probability mass fell inside the reported 95% bounds. Uniform distributions were defined for the relative risks of mortality due to COVID-19 for each comorbidity covering the range described in Supplementary Table 2
2) Age-specific IFR and relative risks of mortality with comorbidities were sampled from the defined distributions
3) Age-distributed IFR for a theoretical population with no comorbidities (IFR^*_a) were computed using the sampled values as follows:

$$IFR_a^* = \frac{IFR_{h=China,a}}{\sum_i (r_i . c_{i,h,a})} \quad \text{Equation 1}$$

Where $IFR_{h=China,a}$ was the sampled IFR in setting h (where h is China), i is the index for each comorbidity, r_i is the sampled relative risk of mortality for each condition and $c_{i,h,a}$ is the prevalence of each comorbidity in setting h. All terms except relative risk values were indexed by age-group a assuming that there are no interactions between age and relative risk of death due to comorbidity

4) The adjusted IFR for Malawi were then estimated using Equation 2:

$$IFR_{h=Malawi,a} = IFR_a^* \sum_i (r_i * c_{i,h=Malawi,a}) \quad \text{Equation 2}$$

5) Steps 2-4 were repeated 1000 times and the median adjusted IFR and uncertainty intervals were calculated as the 50th, 2.5th and 97.5th quantiles from the sampled estimates

A summary for the average IFR for Malawi was obtained by weighting the age-specific IFR by the proportion of the population in each age-group, as follows:

$$IFR_{h=Malawi} = \frac{\sum_a (N_a . IFR_{h=Malawi,a})}{\sum_a N_a} \quad \text{Equation 3}$$

Where N_a is the number of persons in that age-group. The analysis was repeated using data on IFR from Brazil to determine whether the choice of primary data would affect the predicted IFR in Malawi.

For peer review only

This method assumed that the differences in IFR between settings are driven by the presence of comorbidities and differences in age-structure of the population.

ii) Predicted IFR adjusting for health system constraints

The effect of access to and quality of healthcare was accounted for through the following additional steps:

1) Parameter sets defining the proportion of COVID-19 cases requiring different levels of hospital care (severe or critical) were generated using rejection sampling on the basis of (i) prior information from high-income settings and (ii) assumptions for the mortality according to disease severity with treatment (Supplementary Table 3)
2) These estimates of disease severity were used in the simulation model (detailed in the following section) to simulate epidemic trajectories over 365 days without non-pharmaceutical interventions where the availability of hospital care was limited to the level currently prevailing in Malawi.
3) The induced overall IFR in Malawi given the current healthcare system constraints at the end of the epidemic was "Number died of COVID-19 / Number ever infected with SARS-CoV-2".

Further details are provided in the SI.

The resulting population-level IFR predictions for Malawi were compared with China using Monte Carlo simulation. IFR values for China were sampled 1000 times from the lognormal distributions for comparison and the Kolmogorov-Smirnov (KS) test statistic was computed, producing a KS test statistic distribution.

2. Estimates of the potential impact of NPI on transmission of SARS-CoV-2

The COVID-19 Model of Walker *et al.* was used to make projections of the spread of SARS-CoV-2 in Malawi under a number of NPI scenarios.(26) Briefly, the deterministic model comprises an age-structured SEIR compartmental framework which describes the transmission of SARS-CoV-2 through an otherwise homogenous population. The contact rates between age-groups were derived from the Manicaland study in Zimbabwe (Supplementary Table 4).(27) Infected (and infectious) cases were classified as mild (not requiring

For peer review only

care), severe (requiring hospitalisation / oxygen) and critical (requiring ICU / mechanical ventilation) with the likelihood of receiving care constrained by the prevailing health system capacity. We assumed that not all critical cases required mechanical ventilation, but for those that do, access to an ICU bed also indicates availability of mechanical ventilation. In each infected stage, there was a probability of death, derived from multinational analyses from data in China, UK and US. It was assumed that hospitalised cases would not contribute to transmission. In the case of a person needing care but health system capacity being exhausted, the person was exposed to a risk of death consistent with no care being received (Supplementary Table 5).

Epidemic setting

We assumed $R_t = 2$, which was a central value for the estimates of R_t in Malawi immediately prior to the second wave and varied this between 1.5 and 2.5 to reflect the uncertainty in this assumption.(28) At the start of the simulation, 20 cases were seeded in age-groups 35-54 reflecting the ages of those who are most likely to be working or travelling and acquired infection. The population was considered to be fully susceptible during this simulated second wave, due to waning immunity following infection in the first wave along with the emergence of newer strains capable of evading pre-existing immune responses.(29)

Non-pharmaceutical intervention strategies

The NPIs that were in place at the start of the second wave are summarised in Table 1. We additionally consider the potential impact of shielding those aged ≥ 60 years and lockdown. The duration of lockdown was varied between 6 and 24 weeks, whereupon the previously implemented intervention strategies were resumed. The trigger day for the implementation of NPI was when the rate of deaths exceeded 1.0 deaths per 100,000 population per week.

Face coverings were analysed as an incremental intervention on top of the existing measures and we explored a full range of values for efficacy and proper usage assuming that the current measures in place remained for the duration of the simulation. We did not distinguish between household and non-household transmission and assumed adherence and efficacy jointly reduced the risk of transmission to the whole population.

For peer review only

The low numbers of deaths reported to date in Malawi coupled with the high potential for under-reporting make formal calibration to surveillance data problematic. We opted, therefore, to present a hypothetical scenario, using plausible estimates for transmission rates in this setting and incorporating uncertainty around key assumptions.

We ran each simulation 1000 times over 365 modelled days using the sampled parameter sets for disease severity (see above). The outcomes of each intervention on the daily number infected, health system requirements (broken down by severity) and deaths are presented.

3. Estimates of the impact of increasing health system capacity

The projected impact of increasing the number of non-intensive care hospital beds plus availability of oxygen and the introduction of a novel therapeutic agent were examined assuming that the current intervention strategies would remain in place indefinitely. We simulated an increase in hospital bed capacity (plus access to oxygen) by up to 100% from the trigger day of when the rate of deaths exceeded 1.0 deaths per 100,000 population per week, presenting the resulting impact on the cumulative number of deaths projected to occur over the epidemic. Additionally, the impact of a novel therapeutic agent was analysed, assuming a proportional reduction in mortality for severe and critical cases (0.65 and 0.8 respectively, which is of the same order of magnitude as indicated in the RECOVERY trial on dexamethasone, but the modelled agent is hypothetical and not dexamethasone) applicable to all age-groups.(17) We assumed that the therapeutic agent could be administered to those in need even if hospital beds or ICU beds were not available.

All analyses were conducted in R statistical software, version 3.6.3 (https://www.r-project.org/).

Data sharing agreement

Source code and supporting documentation for the SEIR model is available at https://github.com/mrc-ide/squire. All data used in the analyses are publicly available and sources have been listed for each.

For peer review only

Patient and Public Involvement

Patients and/or the public were not involved in the design, or conduct, or reporting, or dissemination plans of this research.

Ethical Approval

Ethical approval was not required for this study which uses publicly available, anonymised data.

Role of the funding source

The funders of this study had no role in study design, data analysis, data interpretation or writing of the report. All authors had access to all data in the study and accept final responsibility for the decision to submit for publication.

For peer review only

RESULTS

1. Predictions of infection fatality ratios in Malawi

Under the assumption of similar healthcare availability, the predicted age-specific IFR for Malawi are higher for every age-group than those reported in China. However, the predicted population-weighted IFR is lower (IFR 0.26%, 95% uncertainty interval [UI] 0.12 – 0.69%, compared with 0.6%, 95% confidence interval 0.40% – 1.30% in China) due to the younger average age of the population (Figure 1). Incorporating health system constraints through the simulation model results in significantly higher age-specific IFR for Malawi (P<0.05, KS test for all age-groups), although the population-weighted estimate is not significantly different to that reported for China (overall IFR 0.83%, 95% UI 0.49% – 1.39%). When using the Brazil data, the adjusted population-weighted IFR for Malawi assuming no health system constraints is 0.48% (95% uncertainty interval [UI] 0.33 – 0.64%), compared with 0.26% (95% UI 0.12 – 0.69%) when using the Chinese data (Supplementary Figure 2).

2. Estimates of the potential impact of NPI on transmission of SARS-CoV-2

The projected unmitigated scenario is presented as a counterfactual, showing what could occur had no interventions been introduced (Figure 2). With the current interventions in place, and assumed to be in place indefinitely, we estimate approximately 54,400 deaths (95% UI 26,900 – 97,300) could be averted over the course of the epidemic compared with an unmitigated scenario in which 134,300 deaths (95% UI 82,100 – 222,500) are projected to occur (Table 2). Enhanced shielding of people aged ≥ 60 years could avert a further 40,200 deaths (95% UI 25,300 – 69,700) and halve ICU admissions at the peak of the outbreak. These measures would also delay the spread of infection, shifting the peak in infections by approximately 66 days.

The predicted age distribution of infected people at the peak of the epidemic shows that the majority of infections occur in the younger ages (<20 years) which make up >50% of the population and have high contact rates (Supplementary Figure 3), although only 1.3% of deaths occur in that group. There is, however,

For peer review only - http://bmjopen.bmj.com/site/about/guidelines.xhtml

considerable uncertainty around the prevalence and impact of comorbidities such as HIV and malnutrition in these age-groups. The majority of projected deaths occur in those aged over 70 years.

Of the mitigation strategies modelled, long-term lockdown has the largest impact, bringing the infection rate down to 275 infections per 1,000 population (95% UI 224 – 327 per 1,000 population) and the mortality rate to 1.8 deaths per 1,000 population (95% UI 1.4 – 2.2 per 1,000 population, equivalent to 33,800 deaths, 95% UI 26,600 – 41,900, Table 1). Applying lockdown over 6, 12 and 24 weeks delays the peak incidence, reducing the size of the outbreak and maintaining hospital requirements below capacity, which in turn reduces mortality rates (Supplementary Figure 4). The impact of each intervention with R_t=1.5 and 2.5 are shown in Supplementary Table 6.

With current interventions in place, coverage of face coverings would need to exceed 60% (30% with shielding implemented simultaneously) with a minimum efficacy of 50% in order to reduce the projected R_t to below 1 (Figure 3).

3. Estimates of the impact of increasing health system capacity

Increasing capacity for non-intensive care (general hospital beds with oxygen availability) by 50% reduces the projected mortality rate to 3.7 deaths per 1,000 population (95% UI 2.8 – 5.3) compared with 4.2 (95% UI 2.9 – 6.5) under the current scenario (Supplementary Table 7). Doubling hospital and oxygen capacity could marginally reduce this further to 3.5 deaths per 1,000 population (95% UI 2.8 – 4.7). Introducing a novel therapeutic agent that is capable of reducing mortality by 0.6 for severe cases and by 0.85 for critical cases, in combination with a 50% scale up in hospital capacity reduces projected deaths to 2.5 deaths per 1,000 population (95% UI 1.9 – 3.6, Figure 4).

For peer review only

DISCUSSION

The results shown here give several important insights into the potential spread and severity of SARS-CoV-2 infection in Malawi and what can be done to prepare for it.

First, under the assumption of similar access to healthcare, overall population-weighted IFR are lower than the reported values for China, consistent with findings from Brazeau *et al* (estimates for low-income countries 0.23 95% prediction interval 0.14, 0.42) due to the younger average age of the population.(21, 30) As the availability of health care is much lower in Malawi, the overall expectation for IFR increases to 0.83%, 95% UI 0.49% – 1.39%, within the range of estimates reported across the Americas, Asia and Europe (overall IFR 0.70, 95% CI 0.57 – 0.82, range 0.28 – 0.89).(31) Estimates of mortality in Uganda follow a similar trend, with a lower predicted disease burden than the comparator regions (Europe, America and China) and the majority of risk arising indirectly due to disruptions to the health service.(32) Other studies have also reported a differential risk of severe disease in African settings compared with European countries due to underlying health conditions although there is considerable uncertainty in these analyses.(33, 34)

Second, we find that the intervention strategies that have been implemented so far in Malawi would be unlikely to suppress or substantially mitigate the epidemic. Although lockdowns have been highly effective across a number of settings, they may be impractical in Malawi for a number of reasons.(3, 35, 36) Approximately 50% of the Malawian population lives in poverty, meaning there is no financial buffer if people are unable to earn money.(36, 37) The disruptions to food production and delivery chains are likely to impact those who are most vulnerable, with food shortages likely to occur within days of lockdown being implemented.

We find that, similar to other studies, shielding of the older population (plus other vulnerable populations, such as people living with HIV or TB) would be effective in significantly reducing the death rate.(38, 39) The practicalities of moving elderly people into separate accommodation in the African context are uncertain at best but it is a potentially risky strategy as a single imported case could have devastating effects. In high-income countries, this strategy has proven difficult with outbreaks occurring in many nursing homes.(40) Designating

For peer review only

shielded households, or even single rooms within a household may be a viable strategy in Malawi which has an extensive network of community health workers who could facilitate and support such measures.

Given the difficult choices facing decision-makers in Malawi and elsewhere, it is not surprising that significant attention has turned to less disruptive strategies for restricting some population movement and advocating use of face coverings, a putatively low-cost and readily available means of reducing transmission risk.(41) Delaying the epidemic by even a few months could allow time for new vaccines to be delivered and for further therapeutics to be tested and introduced.(17, 42, 43) Additional interventions, such as testing (test, trace, isolate) and local quarantining could have a significant impact on the spread of infection and have been regularly used for case-finding and containment for HIV and TB in low and middle-income countries.(44-47) However they are extremely intensive and difficult to implement at a large scale and therefore may not be a feasible option for mitigating the outbreak in Malawi.

Third, we find that increases in certain types of capacity in hospital may contribute to reducing deaths. The current priorities for COVID-19 response in Malawi are to distribute vaccines to health workers and significantly expand access to oxygen concentrators.(15, 48) There are likely to be improvements to long-term morbidity and lung health if severe cases receive oxygen when required, combined with reduced probability of requiring mechanical ventilation, although we do not capture this here. There is an urgent need for further data to analyse the longer-term impacts of COVID-19 infection to inform this. Incorporating the predicted impact of a vaccine, prioritised for health workers, is not possible using this model as we do not capture occupation-based exposure risks. WHO strongly recommends systemic corticosteroid therapy (dexamethasone) for severe COVID-19 infections, either alone or in combination with other drugs, which may reduce mortality and the need for mechanical ventilation.(42) The impact of these therapies in sub-Saharan Africa is not yet well documented, and is likely to be affected by co-infections, particularly HIV and TB coupled with late presentation to care.

The potential risk of nosocomial transmission is high, compounded by global shortages in personal protective equipment (PPE).(49) In response, the Ministry of Health in Malawi developed COVID-19 treatment centres

For peer review only

away from central hospitals and developed reusable PPE equipment to supplement those already acquired.(50) We optimistically assume here that hospitalised cases are isolated and do not contribute to onwards transmission either in the community or to healthcare workers, which may bias our estimates of disease spread. Additionally, discounting this risk lowers the expected impact of NPIs. Other modelling studies have shown variable risks in within-hospital transmission, with Evans *et al* suggesting up to 89% of infections in healthcare workers in England were acquired within the health system and Treibel *et al* finding the majority of these infections were acquired through community transmission.(49, 51)

The unadjusted IFR that we use to derive the mortality rates along with some key parameters including treatment outcomes rely on data from high and middle-income settings which may not be directly transferable to Malawi.(3, 21, 26) Our results are sensitive to the assumptions inherent in these analyses and cannot yet be fully parameterised using data from African settings due to the limited numbers of cases there. Age-structured contact matrices are derived from studies in Zimbabwe although we expect that there are unlikely to be significant differences between the two settings that would meaningfully affect the conclusions drawn. Household structure is not incorporated therefore we cannot adjust for increased risk of infection within households. This may be of particular importance in Malawi where households are multigenerational and there may not be space to designate separate rooms for high-risk individuals. An important next step in this analysis would be the integration of the model with geographically-disaggregated surveillance data on testing, deaths, movement and other data which could capture local transmission and potentially open the way to more finely targeted interventions that may maximise epidemic control with lesser disruptions overall.

The estimates of relative risk of mortality with comorbidities are derived mainly from studies in high or upper-middle-income settings and may vary by age although we do not capture this here. The management of comorbidities is likely to differ across settings and so the corresponding risk of mortality with these conditions may vary also. The relative risks of death of the different comorbidities were combined in an additive model, given that the reported hazard ratios used have been adjusted for the presence of other conditions.

For peer review only - http://bmjopen.bmj.com/site/about/guidelines.xhtml

The low numbers of cases reported in the first wave of the epidemic in Malawi could be consistent with a lower R_t than is assumed here or an imperfect surveillance system with low numbers of tests being carried out. Approximately 1,000 deaths have been reported throughout the whole outbreak, although this is likely an under-estimate, with 82% of those occurring in 2021.(48) We estimate here approximately 80,000 deaths may occur if stricter NPI are not introduced, falling to 48,000 if therapeutics effectively moderate mortality rates. Introduction of a vaccine is likely to have a significant impact on the course of the epidemic, and if prioritised to those at highest risk, could substantially reduce the projected number of deaths.

This study has focussed on the effects of NPI and health system capacity in respect of one disease, COVID-19. However imposing lockdown could disrupt routine health services such as the provision of care for HIV, tuberculosis and malaria along with national immunisation programmes, compounding increases in mortality rates. Balancing the competing demands on health versus economic productivity, poverty and education is an extremely difficult decision and we present these projections as a series of hypothetical scenarios which could be used to inform decision-making.

These outputs are not intended to be a forecast of what will happen in Malawi and evidence should be reviewed in the light of continuously evolving surveillance data and combined with detailed analyses on the broader impacts of any potential interventions. Lessons could be learnt from the South African response, which implemented a rapid, phased strategy, successfully delaying the outbreak despite considerable challenges. In addition to physical distancing and restrictions on movement, South Africa capitalised on existing experienced teams of community health workers to conduct active case-finding along with redirecting contact-tracing teams, previously established for TB control, to conduct COVID-19 contact-tracing and monitor quarantine compliance.(45) Clearly lockdown would have the biggest impact on spread of SARS-CoV-2, however in settings where this is not feasible, a combination of interventions such as shielding, face covering, increasing hospital capacity and therapeutic agents could together have a significant impact on mortality.

Declaration of interests

For peer review only

The authors declare no conflicts of interest.

Acknowledgments

Dr Mangal, Prof Hallett, Prof Phillips, Dr Colbourn, Prof Mfutso-Bengo, Mr Revill, Dr Nkhoma and Mr Ng’ambi are supported by UK Research and Innovation as part of the Global Challenges Research Fund, grant number MR/P028004/1

TM, CW, PW, OJW, AG and TBH acknowledge joint Centre funding from the UK Medical Research Council and Department for International Development. Grant reference: MR/R015600/1

We would like to thank the Imperial College COVID-19 Response Team for their feedback and assistance with method development. We would also like to thank the University of Malawi Health Economics and Policy Unit Think Tank members for their support and collaboration in this project.

For peer review only

For peer review only - http://bmjopen.bmj.com/site/about/guidelines.xhtml

Table 1. List of the interventions under consideration along with their implementation in the model.

Strategy	Implementation
**Current situation:**	Assumed to be in place at the start of the outbreak<br>Workplaces are closed, public events are banned, restrictions on gatherings are in place, public transport is reduced<br>Consider these strategies as a bundle equating to a combined reduction in *Rt* of 24% (9)<br>Dates and details of individual non-pharmaceutical interventions are reported by OxCGRT (6)
**Enhanced shielding:**<br>Current situation, plus:<br>Shielding of those aged ≥ 60 years	Reduce contact rates by 60% for populations aged ≥60 years in addition to the reduction in *Rt* above.<br>It is implemented after a trigger is reached$
**Lockdown:**<br>Current situation, plus:<br>Stay at home requirements<br>School closures<br>Enforce social distancing in excepted businesses<br>Prohibit public transportation<br>Prohibit all gatherings outside household	Consider that this bundle equates to a sustained reduction in *Rt* of 42%.(9)<br>It is implemented after a trigger is reached$

$The trigger date for interventions to be applied was when the rate of death exceeded 1·0 COVID-19 deaths per 100,000 population per week

Table 2. Outputs from intervention strategies over 365 days

	Unmitigated	Current situation	Enhanced shielding	Lockdown
Total cases / 1,000	769.7<br>(668.4 – 872.6)	575.3<br>(483.1 – 667.4)	499.8<br>(412.4 – 591.4)	275.0<br>(223.6 – 326.9)
Number hospital beds required at peak	40,700<br>(28,000 – 59,700)	25,700<br>(14,500 – 34,800)	17,300<br>(9,800 – 26,400)	5,700<br>(3,100 – 9,900)
Number ICU beds required at peak	2,600<br>(2,000 – 3,400)	1,200<br>(900 – 1,600)	600<br>(400 – 700)	300<br>(200 – 400)
Total deaths / 1,000	7.0<br>(4.3 – 11.6)	4.2<br>(2.9 – 6.5)	2.1<br>(1.6 – 2.9)	1.8<br>(1.4 – 2.2)

All values are medians of 1000 simulations using the sampled parameter sets for disease severity. Numbers of hospital and ICU beds are rounded to the nearest 100.

For peer review only

List of Figures

Figure 1. Predicted infection fatality ratios for the Malawian population with unconstrained and constrained healthcare (according to current health system capacity) compared with estimates reported from China.

Figure 2. Impact of non-pharmaceutical interventions compared with a baseline (unmitigated) scenario on the daily incidence per 1,000 population (A), the cumulative deaths per 1,000 population (B), the percentage of hospital beds that are required (C) and the number of ICU beds that are required (D). The unmitigated scenario represents the counterfactual situation had no interventions been introduced. The current situation reflects the non-pharmaceutical interventions adopted by Malawi at the start of the second wave. Enhanced shielding refers to reducing contact rates of people aged ≥ 60 years. Lockdown is the adoption of stringent social distancing policies. Further details are presented in Table 1. The trigger date is shown with a vertical grey dashed line. The red horizontal dashed line shows the capacity of the health system for non-intensive care (C). ICU capacity comprises 25 ICU beds and 16 mechanical ventilators.

Figure 3. Impact of face covering (A) and face covering plus enhanced shielding (B) on the total number of deaths per 1,000 population projected to occur over 365 days. The full range of values for % efficacy and % proper use (adherence) are presented. The current interventions are assumed to remain in place. The isoclines (green lines) represent the estimated *Rt* given the efficacy and adherence.

Figure 4. Projected total numbers of deaths per 1,000 people over 365 days with increases in hospital capacity (and oxygen) and a novel therapeutic agent. The points show the median of 1000 simulations, with 2.5th and 97.5th uncertainty intervals represented by the bars.

References

1. World Health Organization. Coronavirus disease (COVID-19): Situation Report - 209. Geneva, Switzerland; 2020 16th August 2020.
2. Ferguson N, Laydon D, Nedjati Gilani G, Imai N, Ainslie K, Baguelin M, et al. Report 9: Impact of non-pharmaceutical interventions (NPIs) to reduce COVID19 mortality and healthcare demand 2020 [Available from: https://www.imperial.ac.uk/media/imperial-college/medicine/sph/ide/gida-fellowships/Imperial-College-COVID19-NPI-modelling-16-03-2020.pdf.

For peer review only

3. Flaxman S, Mishra S, Gandy A, Unwin HJT, Mellan TA, Coupland H, et al. Estimating the effects of non-pharmaceutical interventions on COVID-19 in Europe. Nature. 2020;584:257-61.

4. Cowling BJ, Ali ST, Ng TW, Tsang TK, Li JC, Fong MW, et al. Impact assessment of non-pharmaceutical interventions against coronavirus disease 2019 and influenza in Hong Kong: an observational study. The Lancet Public Health. 2020;5(5):e279-e88.

5. Kucharski AJ, Klepac P, Conlan AJ, Kissler SM, Tang ML, Fry H, et al. Effectiveness of isolation, testing, contact tracing, and physical distancing on reducing transmission of SARS-CoV-2 in different settings: a mathematical modelling study. The Lancet Infectious Diseases. 2020;20(10):1151-60.

6. Oxford COVID-19 Government Response Tracker, Blavatnik School of Government [Internet]. 2020 [cited 15th March 2021]. Available from: https://www.bsg.ox.ac.uk/research/research-projects/covid-19-government-response-tracker.

7. National Statistical Office, ICF. Malawi Demographic and Health Survey 2015–2016 2017 [Available from: https://dhsprogram.com/pubs/pdf/FR319/FR319.pdf.

8. Davies NG, Kucharski AJ, Eggo RM, Gimma A, Edmunds WJ, Jombart T, et al. Effects of non-pharmaceutical interventions on COVID-19 cases, deaths, and demand for hospital services in the UK: a modelling study. The Lancet Public Health. 2020;5(7):e375-e85.

9. Li Y, Campbell H, Kulkarni D, Harpur A, Nundy M, Wang X, et al. The temporal association of introducing and lifting non-pharmaceutical interventions with the time-varying reproduction number (R) of SARS-CoV-2: a modelling study across 131 countries. The Lancet Infectious Diseases. 2021;21(2):193-202.

10. Karnakov P, Arampatzis G, Kičić I, Wermelinger F, Wälchli D, Papadimitriou C, et al. Data-driven inference of the reproduction number for COVID-19 before and after interventions for 51 European countries. Swiss medical weekly. 2020;150:w20313.

11. Teachout M, Zipfel C. The economic impact of COVID-19 lockdowns in sub Saharan Africa: International Growth Centre; 2020 [Available from: https://www.theigc.org/wp-content/uploads/2020/05/Teachout-and-Zipfel-2020-policy-brief-.pdf.

12. World Health Organization. Advice on the use of masks in the context of COVID-19 Geneva, Switzerland2020 [updated 5 June 2020. Available from: https://apps.who.int/iris/bitstream/handle/10665/332293/WHO-2019-nCov-IPC_Masks-2020.4-eng.pdf?sequence=1&isAllowed=y.

13. The Royal Society. Face masks and coverings for the general public: Behavioural knowledge, effectiveness of cloth coverings and public messaging. London, UK2020 [Available from: https://royalsociety.org/-/media/policy/projects/set-c/set-c-facemasks.pdf?la=en-GB&hash=A22A87CB28F7D6AD9BD93BBCBFC2BB24.

14. Sonenthal PD, Masiye J, Kasomekera N, Marsh RH, Wroe EB, Scott KW, et al. COVID-19 preparedness in Malawi: a national facility-based critical care assessment. The Lancet Global Health. 2020;8:e890-e2.

15. UNICEF. Malawi: COVID-19 Situation Report No. 6 Lilongwe, Malawi2020 [Available from: https://www.unicef.org/malawi/sites/unicef.org.malawi/files/2020-05/UNICEF%20Malawi%20COVID-19%20Situation%20Report_4%20May%202020%20%28004%29.pdf.

16. Patel BK, Kress JP, Hall JB. Alternatives to Invasive Ventilation in the COVID-19 Pandemic. JAMA. 2020;324(1):43-4.

17. Horby P, Lim WS, Emberson J, Mafham M, Bell J, Linsell L, et al. Effect of Dexamethasone in Hospitalized Patients with COVID-19: Preliminary Report. New England Journal of Medicine. 2020;384:693-704.

18. Horby PW, Pessoa-Amorim G, Peto L, Brightling CE, Sarkar R, Thomas K, et al. Tocilizumab in patients admitted to hospital with COVID-19 (RECOVERY): preliminary results of a randomised, controlled, open-label, platform trial. medRxiv. 2021:2021.02.11.21249258.

19. Banerjee A, Pasea L, Harris S, Gonzalez-Izquierdo A, Torralbo A, Shallcross L, et al. Estimating excess 1-year mortality associated with the COVID-19 pandemic according to underlying conditions and age: a population-based cohort study. The Lancet. 2020;395(10238):1715-25.

20. Office for National Statistics. Deaths registered weekly in England and Wales, provisional: week ending 5 June 2020 2020 [Available from: https://www.ons.gov.uk/peoplepopulationandcommunity/healthandsocialcare/conditionsanddiseases.

21. Verity R, Okell LC, Dorigatti I, Winskill P, Whittaker C, Imai N, et al. Estimates of the severity of coronavirus disease 2019: a model-based analysis. The Lancet Infectious Diseases. 2020;20(6):669-77.

For peer review only

22. Palaiodimos L, Kokkinidis DG, Li W, Karamanis D, Ognibene J, Arora S, et al. Severe obesity, increasing age and male sex are independently associated with worse in-hospital outcomes, and higher in-hospital mortality, in a cohort of patients with COVID-19 in the Bronx, New York. Metabolism. 2020;108:154262.
23. Williamson E, Walker AJ, Bhaskaran KJ, Bacon S, Bates C, Morton CE, et al. OpenSAFELY: factors associated with COVID-19-related hospital death in the linked electronic health records of 17 million adult NHS patients. medRxiv. 2020.
24. Boulle A, Davies M-A, Hussey H, Ismail M, Morden E, Vundle Z, et al. Risk factors for COVID-19 death in a population cohort study from the Western Cape Province, South Africa. Clinical infectious diseases. 2020;Online ahead of print.
25. Ssentongo P, Ssentongo AE, Heilbrunn ES, Ba DM, Chinchilli VM. Association of cardiovascular disease and 10 other pre-existing comorbidities with COVID-19 mortality: A systematic review and meta-analysis. PloS one. 2020;15(8):e0238215.
26. Walker PGT, Whittaker C, Watson OJ, Baguelin M, Winskill P, Hamlet A, et al. The impact of COVID-19 and strategies for mitigation and suppression in low- and middle-income countries. Science. 2020;369(6502):413-22.
27. Melegaro A, Del Fava E, Poletti P, Merler S, Nyamukapa C, Williams J, et al. Social contact structures and time use patterns in the Manicaland Province of Zimbabwe. PloS one. 2017;12(1):e0170459.
28. Situation Report for COVID-19: Africa, 2020-08-17 [Internet]. Imperial College London. 2020 [cited 20th August 2020]. Available from: https://mrc-ide.github.io/global-lmic-reports/Africa/.
29. Kupferschmidt K. New mutations raise specter of 'immune escape'. Science. 2021;371(6527):329.
30. Brazeau N, Verity R, Jenks S, Fu H, Whittaker C, Winskill P, et al. Report 34: COVID-19 infection fatality ratio: estimates from seroprevalence. 2020.
31. Meyerowitz-Katz G, Merone L. A systematic review and meta-analysis of published research data on COVID-19 infection-fatality rates. medRxiv. 2020.
32. Bell D, Hansen KS, Kiragga AN, Kambugu A, Kissa J, Mbonye AK. Predicting the Impact of COVID-19 and the Potential Impact of the Public Health Response on Disease Burden in Uganda. Am J Trop Med Hyg. 2020;103(3):1191-7.
33. Clark A, Jit M, Warren-Gash C, Guthrie B, Wang HH, Mercer SW, et al. Global, regional, and national estimates of the population at increased risk of severe COVID-19 due to underlying health conditions in 2020: a modelling study. The Lancet Global Health. 2020;8(8):e1003-e17.
34. Diop BZ, Ngom M, Biyong CP, Biyong JNP. The relatively young and rural population may limit the spread and severity of Covid-19 in Africa: a modelling study. BMJ global health. 2020;5(5):e002699.
35. Kraemer MU, Yang C-H, Gutierrez B, Wu C-H, Klein B, Pigott DM, et al. The effect of human mobility and control measures on the COVID-19 epidemic in China. Science. 2020;368(6490):493-7.
36. Nachega JB, Grimwood A, Mahomed H, Fatti G, Preiser W, Kallay O, et al. From Easing Lockdowns to Scaling Up Community-based Coronavirus Disease 2019 Screening, Testing, and Contact Tracing in Africa—Shared Approaches, Innovations, and Challenges to Minimize Morbidity and Mortality. Clinical Infectious Diseases. 2020;72(2):327-31.
37. Databank [Internet]. The World Bank Group. 2020 [cited 10th July 2020]. Available from: https://data.worldbank.org/country/malawi.
38. Davies NG, Kucharski AJ, Eggo RM, Gimma A, Edmunds WJ, Jombart T, et al. Effects of non-pharmaceutical interventions on COVID-19 cases, deaths, and demand for hospital services in the UK: a modelling study. The Lancet Public Health. 2020;5(7):e375-e85.
39. van Zandvoort K, Jarvis CI, Pearson CAB, Davies NG, Nightingale ES, Munday JD, et al. Response strategies for COVID-19 epidemics in African settings: a mathematical modelling study. BMC Medicine. 2020;18(1):324.
40. Public Health England. Outbreaks in care homes in England 2020 [Available from: https://assets.publishing.service.gov.uk/government/uploads/system/uploads/attachment_data/file/893025/COVID19_Care_Homes_17_June.pdf.
41. Ngonghala CN, Iboi E, Eikenberry S, Scotch M, MacIntyre CR, Bonds MH, et al. Mathematical assessment of the impact of non-pharmaceutical interventions on curtailing the 2019 novel Coronavirus. Mathematical Biosciences. 2020;325:108364.
42. Siemieniuk R, Rochwerg B, Agoritsas T, Lamontagne F, Leo Y-S, Macdonald H, et al. A living WHO guideline on drugs for covid-19. BMJ. 2020;370:m3379.
43. The RECOVERY Collaborative Group. Dexamethasone in Hospitalized Patients with Covid-19. New England Journal of Medicine. 2020;384(8):693-704.
44. Hellewell J, Abbott S, Gimma A, Bosse NI, Jarvis CI, Russell TW, et al. Feasibility of controlling COVID-19 outbreaks by isolation of cases and contacts. The Lancet Global Health. 2020;8(4):e488-e96.

For peer review only

45. Abdool Karim SS. The South African Response to the Pandemic. New England Journal of Medicine. 2020;382:e95.

46. Shapiro AE, Variava E, Rakgokong MH, Moodley N, Luke B, Salimi S, et al. Community-based targeted case finding for tuberculosis and HIV in household contacts of patients with tuberculosis in South Africa. American journal of respiratory and critical care medicine. 2012;185(10):1110-6.

47. Reddy KP, Shebl FM, Foote JH, Harling G, Scott JA, Panella C, et al. Cost-effectiveness of public health strategies for COVID-19 epidemic control in South Africa: a microsimulation modelling study. The Lancet Global Health. 2021;9(2):e120-e9.

48. UNICEF. MALAWI COVID-19 Situation Report, 17 February 20212021 15th March 2021. Available from: https://www.unicef.org/media/94356/file/Malawi-COVID19-SitRep-17-February-2021.pdf.

49. Evans S, Agnew E, Vynnycky E, Robotham J. The impact of testing and infection prevention and control strategies on within-hospital transmission dynamics of COVID-19 in English hospitals. medRxiv. 2020:2020.05.12.20095562.

50. Chaziya J, Freyne B, Lissauer S, Nielsen M, Langton J, Hare B, et al. COVID-19 in Malawi: lessons in pandemic preparedness from a tertiary children's hospital. Archives of Disease in Childhood. 2021;106(3):238.

51. Treibel TA, Manisty C, Burton M, McKnight Á, Lambourne J, Augusto JB, et al. COVID-19: PCR screening of asymptomatic health-care workers at London hospital. The Lancet. 2020;395(10237):1608-10.

For peer review only

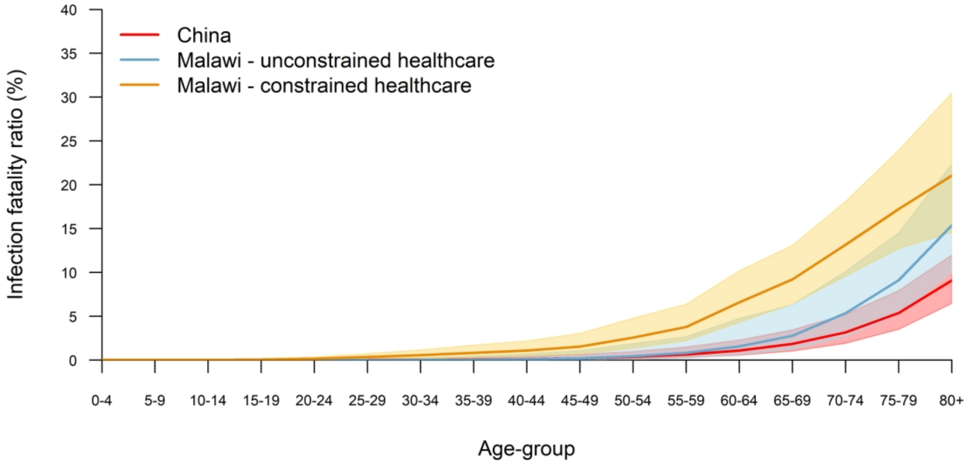

Figure 1. Predicted infection fatality ratios for the Malawian population with unconstrained and constrained healthcare (according to current health system capacity) compared with estimates reported from China.

203x101mm (300 x 300 DPI)

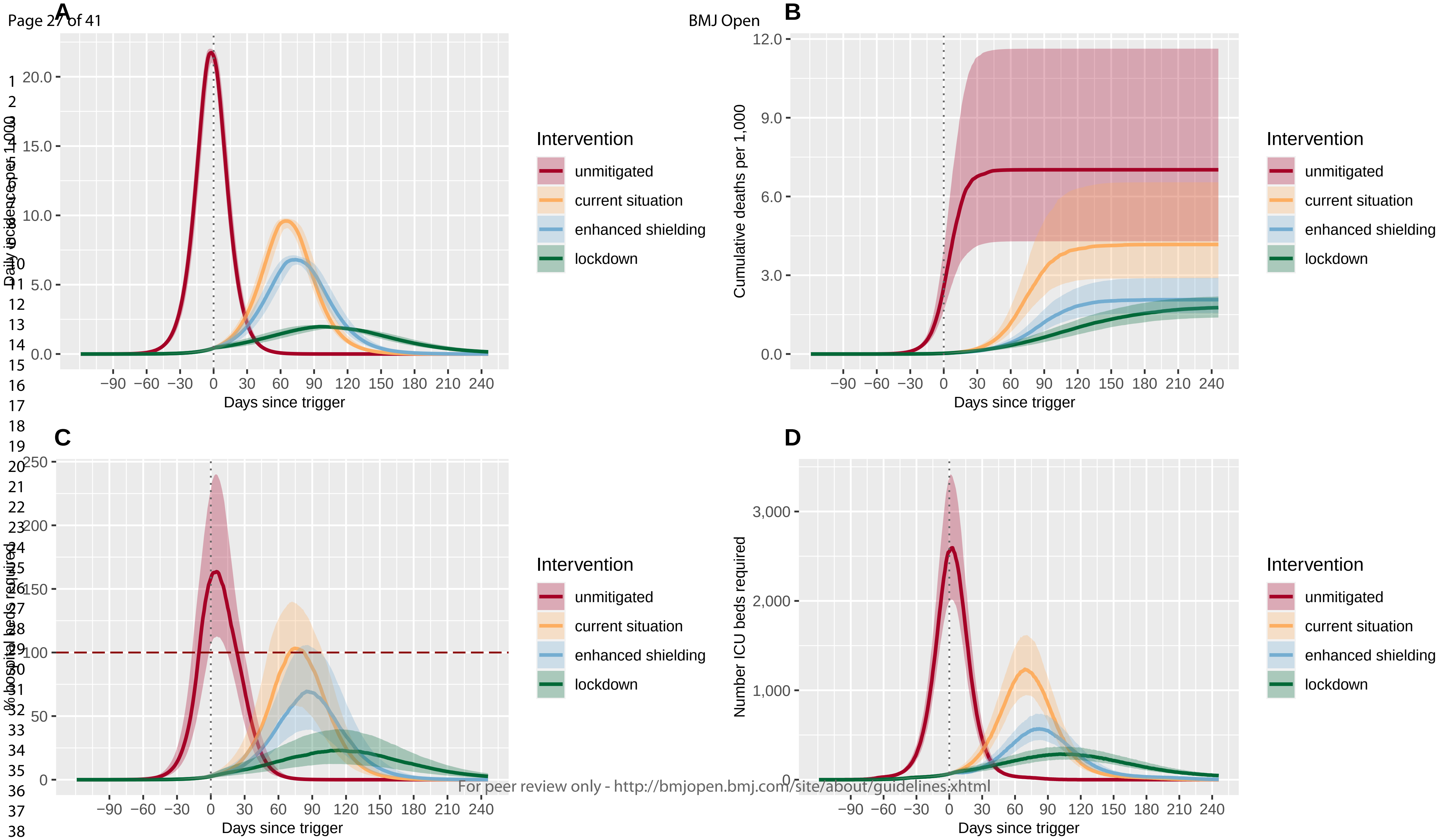
A
Daily incidence per 1,000
20.0
15.0
10.0
5.0
0.0
−90 −60 −30 0 30 60 90 120 150 180 210 240
Days since trigger
Intervention
unmitigated
current situation
enhanced shielding
lockdown
B
Cumulative deaths per 1,000
12.0
9.0
6.0
3.0
0.0
−90 −60 −30 0 30 60 90 120 150 180 210 240
Days since trigger
Intervention
unmitigated
current situation
enhanced shielding
lockdown
C
% hospital beds required
250
200
150
100
50
0
−90 −60 −30 0 30 60 90 120 150 180 210 240
Days since trigger
Intervention
unmitigated
current situation
enhanced shielding
lockdown
D
Number ICU beds required
3,000
2,000
1,000
0
−90 −60 −30 0 30 60 90 120 150 180 210 240
Days since trigger
Intervention
unmitigated
current situation
enhanced shielding
lockdown

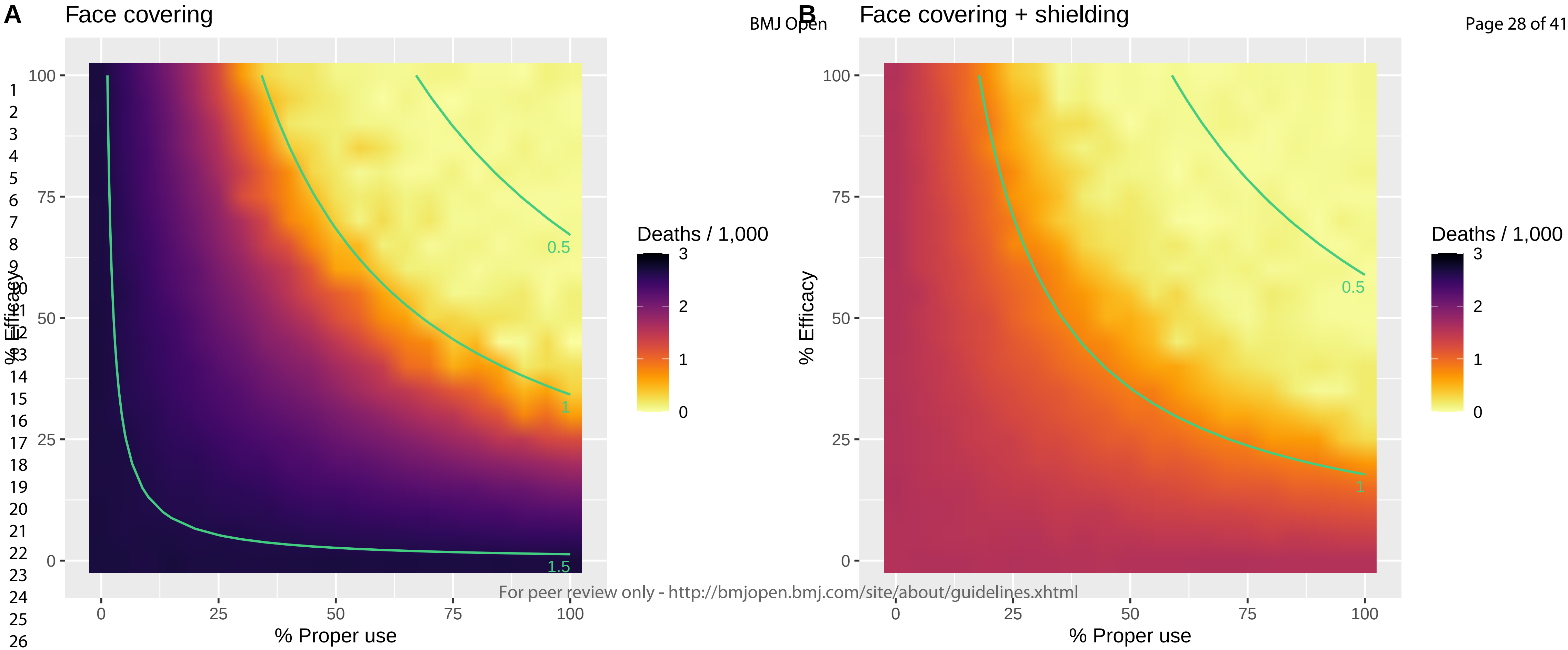
A
Face covering
B
Face covering + shielding
% Efficacy
% Proper use
0
25
50
75
100
Deaths / 1,000
3
2
1
0
1.5
1
0.5

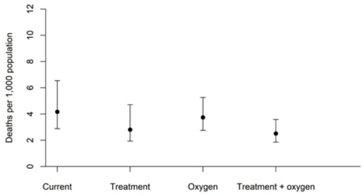

Intervention	Notes
Current	Assumes current interventions remain in place
Treatment	Current interventions in place plus availability of a therapeutic agent for all severe / critical cases (irrespective of hospital bed availability)
Oxygen	Current interventions in place plus 50% scale-up in hospital beds and oxygen availability
Treatment + oxygen	Current interventions in place plus 50% scale-up in hospital beds and oxygen availability and availability of a therapeutic agent for all severe / critical cases (irrespective of hospital bed availability)

Figure 4. Projected total numbers of deaths per 1,000 people over 365 days with increases in hospital capacity (and oxygen) and a novel therapeutic agent. The points show the median of 1000 simulations, with 2.5th and 97.5th uncertainty intervals represented by the bars.

338x190mm (96 x 96 DPI)

Supplementary Information

<u>Prevalence of comorbidities in Malawi and China</u>

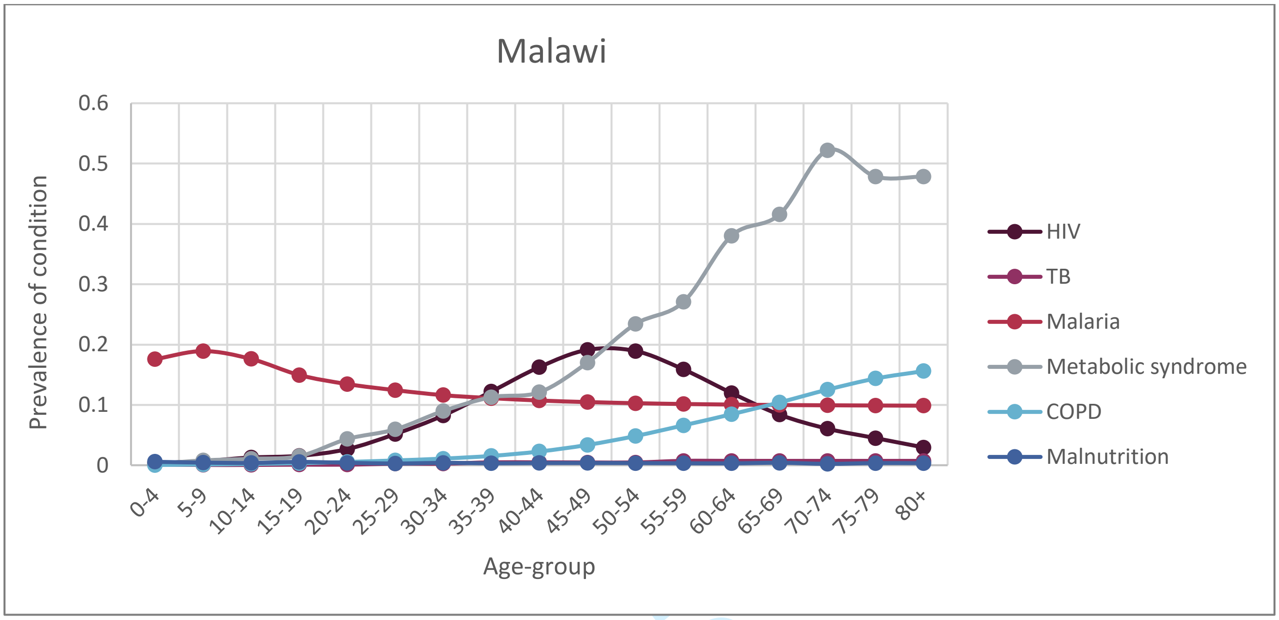

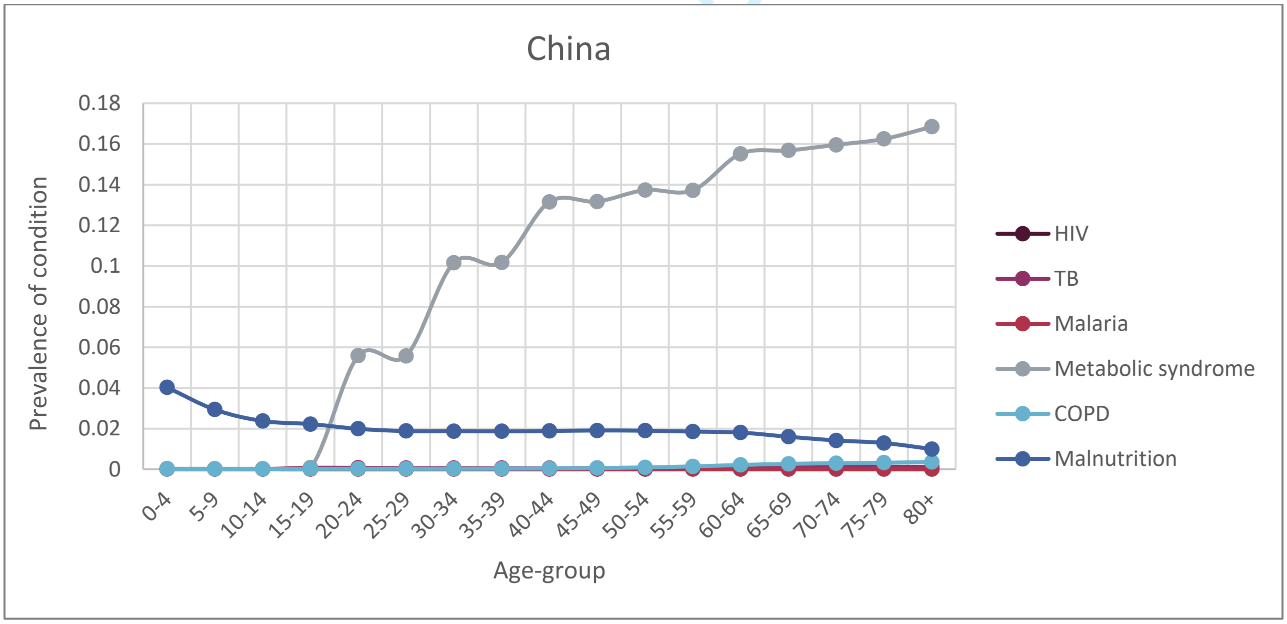

Supplementary Figure 1. Prevalence of key diseases in Malawi (top figure) and China (lower figure) used for the estimation of country-specific infection fatality ratios. Metabolic syndrome, defined in the main text and Supplementary Table 2, is a composite measure of four chronic diseases which tend to cluster in individuals.

Supplementary Table 1. Prevalence data used for the calculations of disease severity and infection fatality ratios.

Data	Source	Notes
TB	WHO TB Report (1)	The estimated incidence of active TB in 2019: number of active cases divided by population size for every age-group and by sex
HIV	UNAIDS Country Projections 2019 (2)<br><br>IHME (3)	For Malawi: includes all PLHIV, irrespective of treatment status. Projections are from 2019<br><br>For China and Brazil: includes all PLHIV, irrespective of treatment status, data are from 2019
Malaria	IHME (3)	For Malawi and Brazil only. Prevalence of clinical malaria episodes in 2019.
COPD	IHME (3)	For all countries, data from 2019
CVD	IHME (3)	For all countries, data from 2019<br>Included as part of "metabolic syndrome"
Diabetes	Price *et al* (4)<br><br>IHME (3)	Included as part of "metabolic syndrome"<br>Price et al 2018 for Malawi estimates and IHME (2019) for China / Brazil estimates
Hypertension	Price *et al* (4)	Included as part of "metabolic syndrome"<br>Price et al 2018 for Malawi estimates. Hypertension is included in the CVD prevalence estimates for China and Brazil.
Obesity	Global Obesity Observatory (5)	Included as part of "metabolic syndrome"<br>Malawi data are from 2016, China data are from 2015-2019, Brazil data are from 2013-2014
Malnutrition	IHME (3)	For all countries, data from 2019
Population size	UNdata (6)	For all countries, data from 2019

All disease prevalence estimates are age- and sex-stratified.

For peer review only

Supplementary Table 2. Parameters used in the simulation model

Parameter	Value	Notes / source
R_t	2	range 1.5-2.5
Hospital bed capacity	1.3 beds per 1,000 population	Equates to 24,869 hospital beds. Source: World Bank (7)
ICU bed capacity	25	
Mean latent period	4.6 days	Estimated at 5.1 days. The last 0.5 days are incorporated in the infectious periods to capture pre-symptomatic infectivity
Mean duration of mild infection	2.1 days	Incorporates 0.5 days of infectiousness prior to symptoms. In combination with mean duration of severe illness this gives a mean serial interval of 6.75 days
Mean duration of severe infection prior to hospitalisation	4.5 days	Mean onset-to-admission of 4 days based on unpublished analysis of data from the ICNARC study. Includes 0.5 days of infectiousness prior to symptom onset
Mean duration of hospitalisation for non-critical cases if survive	9.5 days	Based on unpublished analysis of data from the ICNARC study
Mean duration of hospitalisation for non-critical cases if die	7.6 days	Based on unpublished analysis of data from the ICNARC study
Mean duration in ICU if survive	11.3 days	Based on data from the ICNARC study adjusted for censoring
Mean duration in ICU if die	10.1 days	Based on data from the ICNARC study adjusted for censoring.
Mean duration in recovery after ICU	3.4 days	Based on unpublished analysis of data from the ICNARC study
Relative risk of mortality for patients with HIV*	1.70 – 2.70	Samples are drawn from the uniform distribution using the range defined as lower and upper bounds (8)
Relative risk of mortality for patients with TB	1.81 – 4.04	Samples are drawn from the uniform distribution using the range defined as lower and upper bounds (8)
Relative risk of mortality for patients with malaria	1.0 – 3.0	Assumed values. Samples are drawn from the uniform distribution using the range defined as lower and upper bounds
Relative risk of mortality for patients with COPD	0.77 – 4.24	Samples are drawn from the uniform distribution using the range defined as lower and upper bounds (9, 10)
Relative risk of mortality for patients with malnutrition	1.0 – 3.0	Assumed values. Samples are drawn from the uniform distribution using the range defined as lower and upper bounds
Relative risk of mortality for patients with “metabolic syndrome”	1.02 – 5.26	Samples are drawn from the uniform distribution using the range defined as lower and upper bounds. The range is defined using the lower and upper limits from the estimated values below.
Relative risk of mortality for patients with cardiovascular disease	1.20 – 5.26	Used to inform the combined “metabolic syndrome” risk (9, 10)
Relative risk of mortality for patients with hypertension	1.09 – 1.57	Used to inform the combined “metabolic syndrome” risk (8)
Relative risk of mortality for patients with obesity	1.10 – 4.34	Used to inform the combined “metabolic syndrome” risk (9)
Relative risk of mortality for patients with diabetes	1.02 – 2.84	Used to inform the combined “metabolic syndrome” risk (9, 10)

15 *Relative risks for HIV refer to unsuppressed and suppressed HIV infections. Where references are not cited,

16 we use the default parameters from the COVID-19 Model of Walker *et al*.(11)

17

For peer review only

Estimates of disease severity

Following Walker *et al.*, we distinguish three levels of disease severity for COVID-19: (i) those that do not require hospitalisation; (ii) those that do require hospitalisation but not intensive care; (iii) those that require intensive care (ICU).(11) Two parameters govern the proportions in each category: the proportion of infected cases requiring hospitalisation (*p_severe*) and the proportion of hospitalised cases requiring ICU (*p_critical*). We use the distributions of sampled adjusted IFR for Malawi, detailed in Methods Section 1 (i) which assume no health system constraints, as bounds for rejection sampling to determine reasonable values for these two parameters.

We sampled from a credible range for each parameter (detailed in Supplementary Table 3) based on values observed in high-income settings, keeping the age-distribution consistent with that reported in Verity *et al* and Walker *et al*.(11, 12) As we have based the calculation of IFR so far on data from China, we assumed that all persons that needed hospitalisation or intensive care received it, and that the proportion of those requiring intensive care that die, *m*, is 50%. This is a generalised assumption which incorporates both the availability of treatment and the mortality rates across both the untreated and treated critical cases. We assume no mortality in severe cases in this instance, although this assumption is changed in the simulation model. Combining the assumptions together, gives an overall IFR across the 17 age-groups as follows, where $prop_a$ is the proportion of the population in age-group *a*:

$$IFR_h = \sum_{a=1}^{17} \left(p\_severe_{h,a} * p\_critical_{h,a} * m\right) * prop_a$$

Parameter sets for *p_severe* and *p_critical* were accepted if the resulting overall IFR fell within the 95% uncertainty interval of the adjusted IFR for Malawi and the process was repeated until 1000 accepted parameter sets were generated. The resulting parameter sets were used as inputs to the epidemic simulation model.

For peer review only

Supplementary Table 3. Estimates of disease severity for the Malawian population

**Age-group (years)**	**Proportion cases requiring hospitalisation**		**Proportion hospitalised cases requiring ICU**	
	Credible range	Median accepted values (2.5th and 97.5th quantiles) *	Credible range	Median accepted values (2.5th and 97.5th quantiles) *
0-4	0.0002-0.0050	0.0013 (0.0005 – 0.0029)	0.0100-0.2500	0.0636 (0.0222 – 0.1456)
5-9	0.0002-0.0050	0.0013 (0.0005 – 0.0029)	0.0100-0.2500	0.0636 (0.0222 – 0.1456)
10-14	0.0002-0.0050	0.0013 (0.0005 – 0.0029)	0.0100-0.2500	0.0636 (0.0222 – 0.1456)
15-19	0.0004-0.0100	0.0027 (0.0010 – 0.0058)	0.0100-0.2500	0.0636 (0.0222 – 0.1456)
20-24	0.0010-0.0250	0.0067 (0.0026 – 0.0145)	0.0100-0.2500	0.0636 (0.0222 – 0.1456)
25-29	0.0020-0.0500	0.0134 (0.0051 – 0.0290)	0.0100-0.2500	0.0636 (0.0222 – 0.1456)
30-34	0.0032-0.0800	0.0215 (0.0082 – 0.0464)	0.0100-0.2500	0.0636 (0.0222 – 0.1456)
35-39	0.0046-0.1150	0.0309 (0.0118 – 0.0668)	0.0106-0.2650	0.0674 (0.0236 – 0.1543)
40-44	0.0058-0.1450	0.0389 (0.0148 – 0.0842)	0.0120-0.3000	0.0763 (0.0267 – 0.1747)
45-49	0.0078-0.1950	0.0523 (0.0200 – 0.1132)	0.0150-0.3750	0.0954 (0.0334 – 0.2183)
50-54	0.0116-0.2900	0.0778 (0.0297 – 0.1684)	0.0208-0.5200	0.1323 (0.0463 – 0.3028)
55-59	0.0144-0.3600	0.0966 (0.0369 – 0.2090)	0.0298-0.7450	0.1895 (0.0663 – 0.4338)
60-64	0.0204-0.5100	0.1369 (0.0522 – 0.2961)	0.0448-1.1200	0.2849 (0.0996 – 0.6521)
65-69	0.0234-0.5850	0.1570 (0.0599 – 0.3396)	0.0614-1.5350	0.3905 (0.1366 – 0.8938)
70-74	0.0292-0.7300	0.1960 (0.0747 – 0.4238)	0.0772-1.9300	0.4909 (0.1717 – 1.0000)
75-79	0.0354-0.8850	0.2376 (0.0906 – 0.5138)	0.0922-2.3050	0.5863 (0.2051 – 1.0000)
80+	0.0360-0.9000	0.2416 (0.0921 – 0.5225)	0.1418-3.5450	0.9018 (0.3154 – 1.000)

* distributions obtained through rejection sampling.

For peer review only

Supplementary Table 4. Social contact matrix between age-groups in Zimbabwe

Age-group	**0-4**	**5-9**	**10-14**	**15-19**	**20-24**	**25-29**	**30-34**	**35-39**	**40-44**	**45-49**	**50-54**	**55-59**	**60-64**	**65-69**	**70-74**	**75+**
**0-4**	1.006	1.030	0.883	0.732	0.647	0.712	0.645	0.463	0.289	0.214	0.211	0.215	0.179	0.121	0.075	0.048
**5-9**	0.807	2.863	2.668	0.968	0.423	0.449	0.468	0.416	0.289	0.212	0.182	0.186	0.177	0.140	0.093	0.066
**10-14**	0.659	2.544	3.766	1.865	0.580	0.413	0.452	0.536	0.375	0.251	0.207	0.208	0.193	0.177	0.136	0.096
**15-19**	0.617	1.041	2.104	2.548	1.070	0.575	0.536	0.645	0.441	0.297	0.267	0.264	0.229	0.225	0.182	0.112
**20-24**	0.947	0.790	1.135	1.858	1.363	0.969	0.752	0.723	0.474	0.363	0.333	0.310	0.281	0.254	0.199	0.123
**25-29**	1.091	0.878	0.848	1.046	1.014	1.131	0.885	0.707	0.425	0.330	0.297	0.299	0.271	0.225	0.153	0.101
**30-34**	1.268	1.175	1.191	1.252	1.010	1.136	1.119	1.032	0.593	0.405	0.356	0.364	0.341	0.249	0.164	0.102
**35-39**	0.976	1.119	1.512	1.612	1.041	0.972	1.105	1.221	0.756	0.485	0.420	0.409	0.358	0.276	0.177	0.105
**40-44**	0.960	1.225	1.668	1.738	1.076	0.923	1.001	1.192	0.863	0.629	0.488	0.434	0.378	0.311	0.207	0.117
**45-49**	0.796	1.007	1.248	1.312	0.922	0.801	0.766	0.856	0.704	0.549	0.446	0.383	0.352	0.298	0.215	0.120
**50-54**	0.844	0.928	1.106	1.266	0.909	0.774	0.722	0.796	0.587	0.479	0.417	0.407	0.397	0.331	0.242	0.159
**55-59**	0.902	0.993	1.163	1.312	0.888	0.818	0.776	0.812	0.547	0.431	0.426	0.483	0.484	0.382	0.281	0.210
**60-64**	0.823	1.041	1.191	1.250	0.882	0.815	0.797	0.781	0.524	0.435	0.457	0.532	0.567	0.449	0.347	0.259
**65-69**	0.695	1.026	1.357	1.534	0.995	0.841	0.725	0.751	0.537	0.459	0.475	0.524	0.560	0.471	0.372	0.333
**70-74**	0.592	0.943	1.442	1.712	1.080	0.791	0.664	0.668	0.495	0.458	0.481	0.532	0.599	0.515	0.455	0.383
**75+**	0.557	0.974	1.485	1.530	0.967	0.763	0.597	0.577	0.406	0.373	0.458	0.579	0.648	0.669	0.556	0.400

Values are estimated number of contacts between persons in each age-group per day. The contact matrix is adjusted to give age-specific contact rates.(13)

For peer review only

Supplementary Table 5. Age-dependent mortality rates in severe and critical cases.

	Severe cases		Critical cases	
Age-group	Probability death with treatment	Probability death no treatment	Probability death with treatment	Probability death no treatment
0-4	0.013	0.6	0.5	0.95
5-9	0.013	0.6	0.5	0.95
10-14	0.013	0.6	0.5	0.95
15-19	0.013	0.6	0.5	0.95
20-24	0.013	0.6	0.5	0.95
25-29	0.013	0.6	0.5	0.95
30-34	0.013	0.6	0.5	0.95
35-39	0.013	0.6	0.5	0.95
40-44	0.015	0.6	0.5	0.95
45-49	0.019	0.6	0.5	0.95
50-54	0.027	0.6	0.5	0.95
55-59	0.042	0.6	0.5	0.95
60-64	0.069	0.6	0.5	0.95
65-69	0.105	0.6	0.5	0.95
70-74	0.149	0.6	0.5	0.95
75-79	0.203	0.6	0.5	0.95
80+	0.580	0.6	0.5	0.95

The probabilities of death are the default values used in the COVID-19 Global Model of Walker *et al*.(11)

Values were derived using data from the ICNARC study in the UK.(14)

For peer review only

Estimates of infection fatality ratios in Malawi

The approach detailed in the main manuscript, using age-specific infection fatality ratios (IFR) from China and adjusting for the demography and comorbidity prevalence in Malawi, was repeated using estimates of IFR from Brazil to determine whether the choice of primary data on which to base this analysis significantly impacted the predicted values for Malawi.(15) The figure below (Supplementary Figure 1) shows the adjusted estimates of IFR by age in a theoretical population with no comorbidities using the data reported from China early in the pandemic and the more recent model estimated values from Brazil which incorporate seroreversion.(12) The median adjusted IFR for a population with no comorbidities is slightly higher when using the Brazil data, although the uncertainty intervals overlap for every age-group. When using the Brazil data, the adjusted population-weighted IFR for Malawi assuming no health system constraints is 0.48% (95% uncertainty interval [UI] 0.33 – 0.64%), compared with 0.26% (95% UI 0.12 – 0.69%) when using the Chinese data.

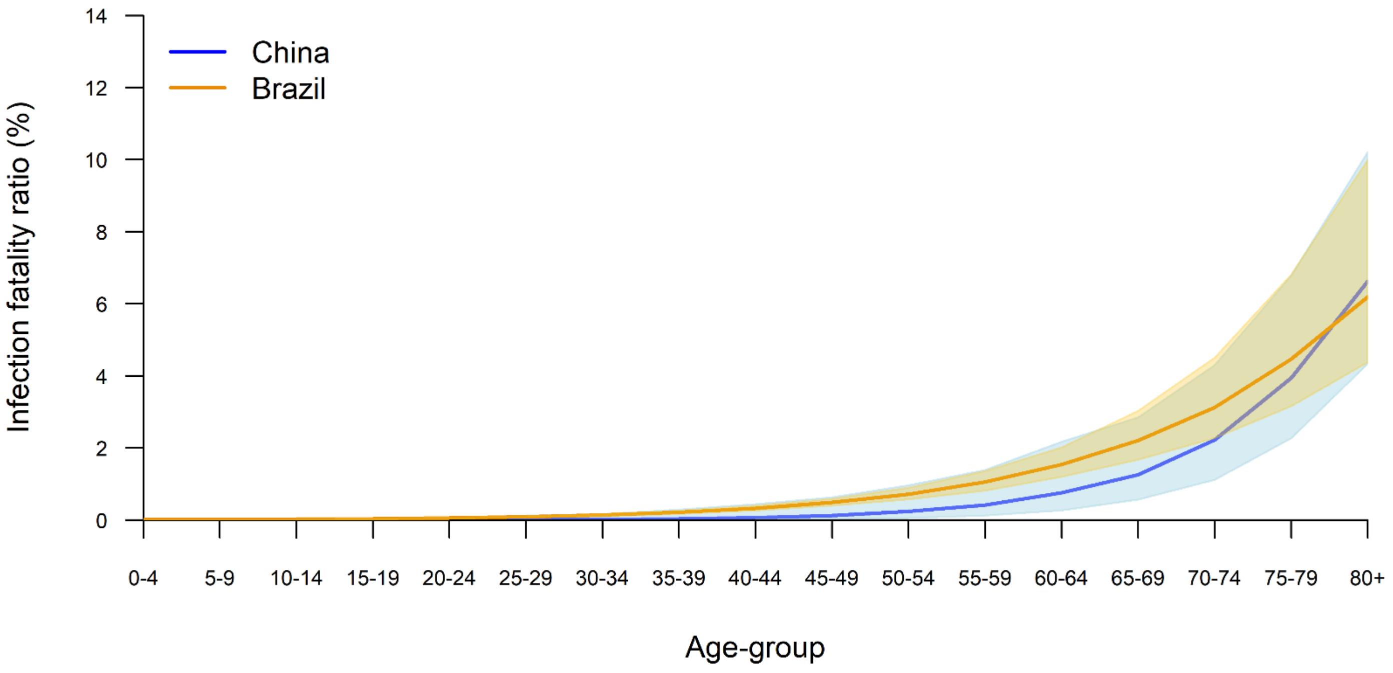

Supplementary Figure 2. Infection fatality ratios in a theoretical population assuming no comorbidities. Estimates are derived from data reported for China (blue) and Brazil (orange).

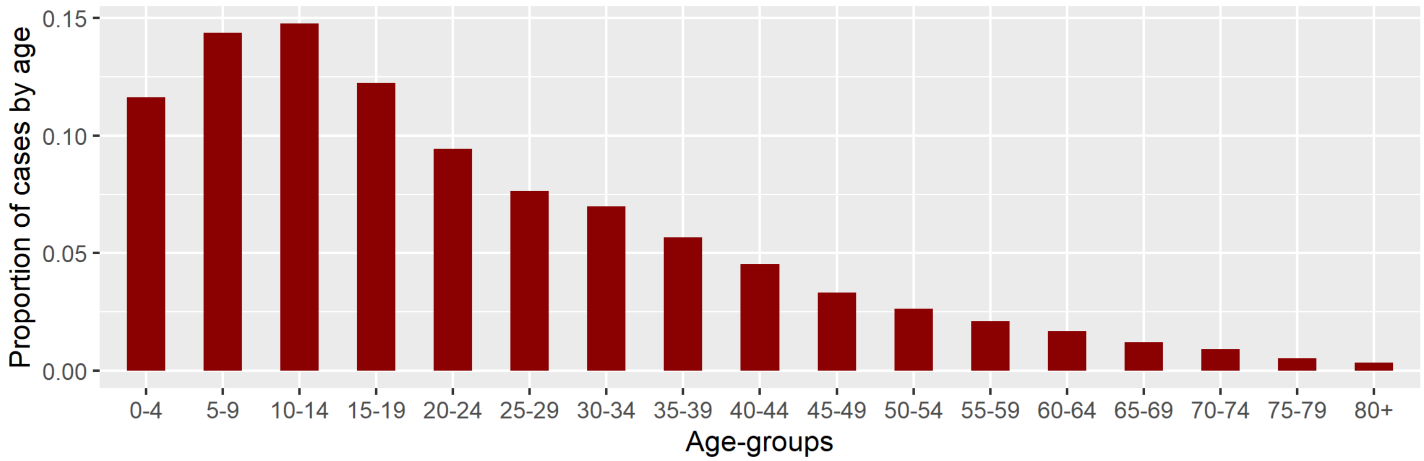

67

68 Supplementary Figure 3. Age distribution of infected cases (asymptomatic and symptomatic) at the peak of the
69 projected unmitigated epidemic.

70

71

72

For peer review only

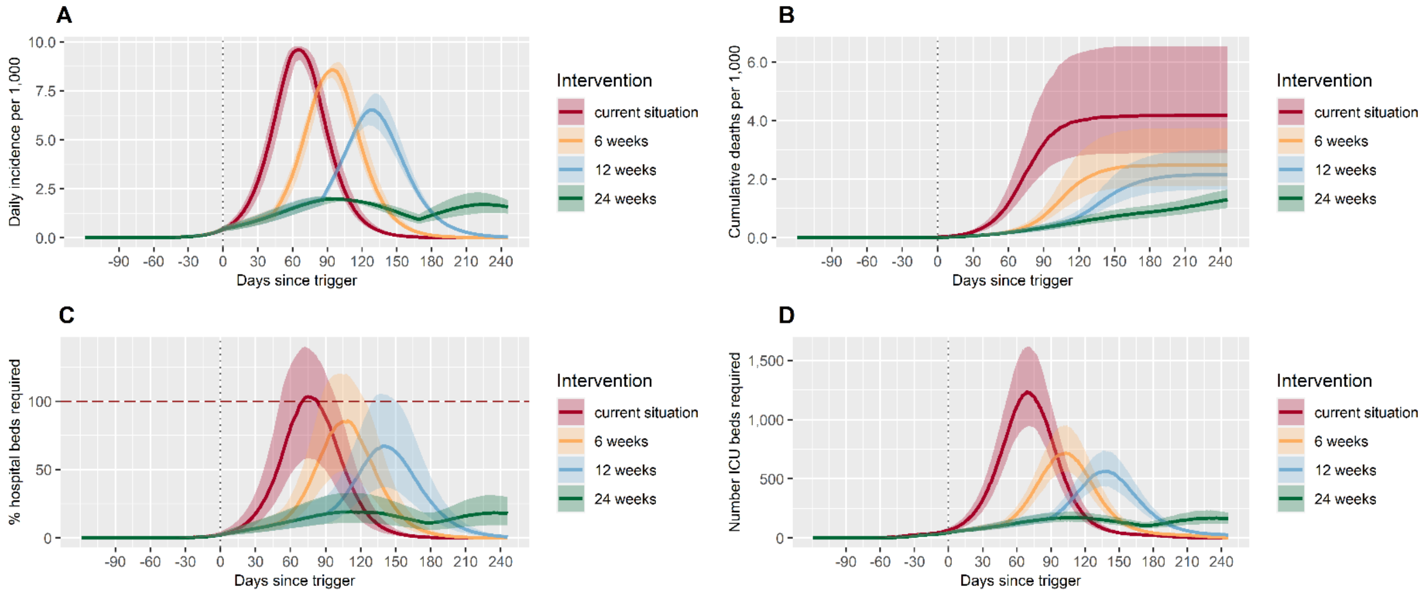

Supplementary Figure 4. Impact of changing the duration of lockdown compared with the current situation on the daily incidence per 1,000 population (A), the cumulative deaths per 1,000 population (B), the percentage of hospital beds that are required (C) and the number of ICU beds that are required (D). The current situation reflects the non-pharmaceutical interventions adopted by Malawi at the start of the second wave. Each intervention represents the implementation of lockdown over 6, 12 or 24 weeks. The trigger date (1 death per 100,000 population per week) is shown with a vertical grey dashed line. The red horizontal dashed line shows the capacity of the health system for non-intensive care (C).

For peer review only

Supplementary Table 6. Simulation outputs under different assumptions of R_0.

		Baseline	Current situation	Enhanced shielding	Lockdown
**R_t = 1.5**	Total cases / 1,000	563.1 (469.8 - 657.9)	21.7 (11 - 33.9)	19.6 (9.6 - 31.7)	18 (9.3 - 28.9)
	Number hospital beds required at peak	25,100 (13,700 – 33,800)	1,000 (500 – 2,000)	700 (300 – 1,400)	700 (300 – 1,400)
	Number ICU beds required at peak	1,200 (900 – 1,500)	100 (100 - 100)	100 (0 - 100)	100 (0 - 100)
	Total number of deaths / 1000	4 (2.8 - 6.3)	0.1 (0.1 - 0.2)	0.1 (0 - 0.2)	0.1 (0 - 0.2)
**R_t = 2.0**	Total cases / 1,000	769.7 (668.4 – 872.6)	575.3 (483.1 – 667.4)	499.8 (412.4 – 591.4)	275.0 (223.6 – 326.9)
	Number hospital beds required at peak	40,700 (28,000 – 59,700)	25,700 (14,500 – 34,800)	17,300 (9,800 – 26,400)	5,700 (3,100 – 9,900)
	Number ICU beds required at peak	2,600 (2,000 – 3,400)	1,200 (900 – 1,600)	600 (400 – 700)	300 (200 – 400)
	Total number of deaths / 1000	7.0 (4.3 – 11.6)	4.2 (2.9 – 6.5)	2.1 (1.6 – 2.9)	1.8 (1.4 – 2.2)
**R_t = 2.5**	Total cases / 1,000	867.8 (764.3 - 972.6)	740.8 (638.8 - 843.1)	689.4 (593.5 - 788.6)	535.5 (458.5 - 613.6)
	Number hospital beds required at peak	53,300 (35,000 – 80,200)	37,900 (26,500 – 54,800)	31,700 (21,600 – 43,000)	23,100 (12,500 – 31,800)
	Number ICU beds required at peak	3,700 (2,900 – 5,000)	2,300 (1,800 – 3,100)	1,300 (1,000 – 1,700)	1,100 (800 – 1,400)
	Total number of deaths / 1000	8.7 (5.4 - 14)	6.5 (4.0 - 10.9)	4.0 (2.6 – 7.0)	3.7 (2.7 - 5.6)

All values are medians of 1000 simulations using the sampled parameter sets. Numbers of hospital beds, ICU beds and deaths are rounded to the nearest 100. The trigger day for interventions (1.0 death per 100,000 population per week) is day 341, 120, and 81 for R_t=1.5, 2.0 and 2.5 respectively.

For peer review only

Supplementary Table 7. Projected cumulative number of deaths with increases in hospital capacity.

**Intervention**	**Total deaths per 1,000 population**
**Current capacity**	4.2 (2.9-6.5)
**Increase capacity by 25%**	3.9 (2.8-5.7)
**Increase capacity by 50%**	3.7 (2.8-5.3)
**Increase capacity by 75%**	3.6 (2.8-5.0)
**Increase capacity by 100%**	3.5 (2.8-4.7)

For peer review only

References

1. WHO TB burden estimates [Internet]. 2020 [cited 2nd March 2021]. Available from: https://www.who.int/tb/country/data/download/en/.
2. AIDSInfo [Internet]. 2020 [cited 22nd May 2020]. Available from: https://aidsinfo.unaids.org/.
3. Global Burden of Disease Study 2019 (GBD 2019) Results [Internet]. Institute for Health Metrics and Evaluation (IHME). 2018 [cited 2nd March 2021]. Available from: http://ghdx.healthdata.org/gbd-results-tool.
4. Price AJ, Crampin AC, Amberbir A, Kayuni-Chihana N, Musicha C, Tafatatha T, et al. Prevalence of obesity, hypertension, and diabetes, and cascade of care in sub-Saharan Africa: a cross-sectional, population-based study in rural and urban Malawi. The Lancet Diabetes & endocrinology. 2018;6(3):208-22.
5. Global Obesity Observatory [Internet]. 2020 [cited 10th June 2020]. Available from: https://www.worldobesitydata.org/.
6. Population by age, sex and urban/rural residence [Internet]. 2020 [cited 22nd May 2020]. Available from: http://data.un.org/Data.aspx?d=POP&f=tableCode%3a22.
7. World Bank Open Data [Hospital beds per 1,000 people] [Internet]. 2020 [cited 16th June 2020]. Available from: https://databank.worldbank.org/home.aspx.
8. Boulle A, Davies M-A, Hussey H, Ismail M, Morden E, Vundle Z, et al. Risk factors for COVID-19 death in a population cohort study from the Western Cape Province, South Africa. Clinical infectious diseases. 2020;Online ahead of print.
9. Noor FM, Islam MM. Prevalence and Associated Risk Factors of Mortality Among COVID-19 Patients: A Meta-Analysis. Journal of Community Health. 2020;45(6):1270-82.
10. Ssentongo P, Ssentongo AE, Heilbrunn ES, Ba DM, Chinchilli VM. Association of cardiovascular disease and 10 other pre-existing comorbidities with COVID-19 mortality: A systematic review and meta-analysis. PloS one. 2020;15(8):e0238215.
11. Walker PGT, Whittaker C, Watson OJ, Baguelin M, Winskill P, Hamlet A, et al. The impact of COVID-19 and strategies for mitigation and suppression in low- and middle-income countries. Science. 2020;369(6502):413-22.
12. Verity R, Okell LC, Dorigatti I, Winskill P, Whittaker C, Imai N, et al. Estimates of the severity of coronavirus disease 2019: a model-based analysis. The Lancet Infectious Diseases. 2020;20(6):669-77.
13. Melegaro A, Del Fava E, Poletti P, Merler S, Nyamukapa C, Williams J, et al. Social contact structures and time use patterns in the Manicaland Province of Zimbabwe. PloS one. 2017;12(1).
14. Intensive Care National Audit & Research Centre. ICNARC report of COVID-19 in critical care2020 1st July 2020. Available from: https://www.icnarc.org/.
15. Brazeau N, Verity R, Jenks S, Fu H, Whittaker C, Winskill P, et al. Report 34: COVID-19 infection fatality ratio: estimates from seroprevalence. 2020.

For peer review only
